# Construction of the porcine genome mobile element variations and investigation of its role in population diversity and gene expression

**DOI:** 10.1186/s40104-024-01121-5

**Published:** 2024-12-04

**Authors:** Jianchao Hu, Lu Gui, Zhongzi Wu, Lusheng Huang

**Affiliations:** National Key Laboratory for Swine Genetic Improvement and Germplasm Innovation, Ministry of Science and Technology of China, Jiangxi Agricultural University, Nanchang, People’s Republic of China

**Keywords:** Gene expression, Genetic diversity, Mobile element variants, Pig, SNP–MEV reference panel

## Abstract

**Background:**

Mobile element variants (MEVs) have a significant and complex impact on genomic diversity and phenotypic traits. However, the quantity, distribution, and relationship with gene expression and complex traits of MEVs in the pig genome remain poorly understood.

**Results:**

We constructed the most comprehensive porcine MEV library based on high-depth whole genome sequencing (WGS) data from 747 pigs across 59 breeds worldwide. This database identified a total of 147,993 polymorphic MEVs, including 121,099 short interspersed nuclear elements (SINEs), 26,053 long interspersed nuclear elements (LINEs), 802 long terminal repeats (LTRs), and 39 other transposons, among which 54% are newly discovered. We found that MEVs are unevenly distributed across the genome and are strongly influenced by negative selection effects. Importantly, we identified 514, 530, and 584 candidate MEVs associated with population differentiation, domestication, and breed formation, respectively. For example, a significantly differentiated MEV is located in the *ATRX* intron between Asian and European pigs, whereas *ATRX* is also differentially expressed between Asian and European pigs in muscle tissue. In addition, we identified 4,169 expressed MEVs (eMEVs) significantly associated with gene expression and 6,914 splicing MEVs (sMEVs) associated with gene splicing based on RNA-seq data from 266 porcine liver tissues. These eMEVs and sMEVs explain 6.24% and 9.47%, respectively, of the observed *cis*-heritability and highlight the important role of MEVs in the regulation of gene expression. Finally, we provide a high-quality SNP–MEV reference haplotype panel to impute MEV genotypes from genome-wide SNPs. Notably, we identified a candidate MEV significantly associated with total teat number, demonstrating the functionality of this reference panel.

**Conclusions:**

The present investigation demonstrated the importance of MEVs in pigs in terms of population diversity, gene expression and phenotypic traits, which may provide useful resources and theoretical support for pig genetics and breeding.

**Supplementary Information:**

The online version contains supplementary material available at 10.1186/s40104-024-01121-5.

## Introduction

Domestic pigs are essential agricultural and medical model animals for humans. Since natural selection and human interference, hundreds of pig breeds with different phenotypic features have been developed worldwide. However, only a small portion of phenotypic variance can be explained by common variations such as single-nucleotide polymorphisms (SNPs) and copy number variants (CNVs) [1, 2]. This prompted us to retrieve lost heritability from more complex repeat sequences, which is known as “genomic dark matter”. Transposons are the most common repeat elements in the pig genome, but they are not necessarily fixed to specific locations owing to mutation, recombination, and unique transposable element (TE) formation mechanisms [3]. Pigs are becoming a promising source for xenotransplantation, with successful progress in the heart and kidney in the past two years [4, 5]. However, pigs carry porcine endogenous retrovirus (PERV), an active mobile element (ME) family, which can potentially spread to humans. As organ transplants from pigs become more common, concerns about the risk of PERV transmission are increasing [6, 7].

MEs, also known as TEs or jumping genes, can move autonomously within the genome. These “jumping genes” are typically prevalent in eukaryotic genomes and can rapidly generate novel genetic variation, increasing genetic diversity and driving genome evolution [3]. In the 1940s, Barbara McClintock published her earliest papers on MEs [8], which earned her the Nobel Prize in 1983. Despite decades of research, there are still many shortcomings in the study of MEVs compared with SNPs, structural variants (SVs) and CNVs. Recent studies have emphasized the significant roles of MEVs in species evolution and environmental adaptation [9]. Furthermore, MEVs are involved in functions such as genome size expansion [10], three-dimensional genome organization [11], chromatin modification [12], gene regulatory networks [13], and even DNA methylation patterns [14].

Despite extensive research on the functions of MEVs in humans, mice, *Drosophila*, and crops [15], few studies have focused on MEVs in livestock. In humans, approximately 60,000 polymorphic MEVs have been identified, some of which are linked to gene expression and complex phenotypes [16]. Livestock also exhibit active and functional MEVs. For example, a long interspersed nuclear element 1 (LINE1, L1) insertion in the *ASIP* gene causes white coat color in swamp buffalo by acting as a potent proximal promoter, increasing *ASIP* transcription tenfold in white buffalo skin [17]. In pigs, mutations in the *VRTN* gene may cause various effects, including increases in vertebral number, carcass length, and teat number [18, 19]. The *VRTN* gene identified in the White Duroc × Chinese Erhualian F2 population in our laboratory has a significant effect on vertebral quantity [20]. Further studies have shown that the first intron of the *VRTN* has a 291 bp element named PRE1-SINE, which may affect the *VRTN* expression [21]. Additionally, the insertion of a SINE into the intron of the porcine growth hormone receptor (*GHR*) gene can reduce its expression by acting as a repressor [22].

Overall, the limited knowledge of MEVs in the pig genome and the lack of large-scale population-level omics data have severely hindered the resolution of MEV-related complex traits in pigs. Here, to comprehensively analyze the distribution and functions of MEVs across the pig genome at the population level, we generated the next-generation sequencing (NGS) data from 747 nonredundant pigs and liver RNA sequencing data from 266 pigs. We created the most comprehensive MEV resource library and SNP–MEV reference haplotype panel and discovered a series of MEVs related to population differentiation and variety formation. Using the liver RNA sequencing data from 266 hybrid pigs, we revealed the relationship between MEVs and gene expression, and recovered part of the “missing heritability”. Finally, by combining the gene expression data of 34 tissues from pig genotype-tissue expression (PigGTEx) [23] and public genome-wide association study (GWAS) data [24], we identified candidate MEVs that affect traits such as total teat number. This study aims to comprehensively investigate and determine the quantity and distribution of MEVs in the pig genome and their relationships with evolutionary selection, gene expression, and complex traits. This study provides important resources and theoretical support for pig genetic research and breeding strategies.

## Methods

### Animal collection

The Ethics Committee of Jiangxi Agricultural University specifically approved this study. All animal work was conducted in accordance with the guidelines for the care and use of experimental animals formulated by the Chinese Ministry of Agriculture. We collected WGS data from 747 nonredundant individuals (Table S1): 380 samples were uploaded and provided in our previous studies [25–30], and 292 samples were from the sixth generation (F6) of the mosaic population, with 266 corresponding RNA sequencing datasets of liver tissue. All F6 animals were born and reared under standardized and consistent housing and feeding conditions at the experimental farm belonging to the National Key Laboratory for Swine Genetic Improvement and Germplasm Innovation, located at Jiangxi Agricultural University in Nanchang, Jiangxi Province, China.

### Data processing

A standard phenol-chloroform extraction protocol was followed to extract genomic DNA from pig ears or muscles. The DNA of each sample was subsequently fragmented into segments ranging from 300 to 500 bp. The NGS library was subsequently constructed according to the standard protocol for library preparation kits. Finally, genome sequencing was performed on the Illumina HiSeq or MGISEQ-2000 platforms to generate paired-end reads of 150 bp, as specified by the manufacturer’s standard protocol.

We filtered out reads that exhibited ≥ 10% missing (“N”) bases or a quality scores of ≤ 20 for ≥ 50% of their bases by using Fastp v0.20.0 [31]. All the cleaned reads were subsequently aligned to the *Sus scrofa* reference genome version 11.1 (Sscrofa11.1) [32] using BWA v0.7.17 [33] (employing the BWA-MEM algorithm with default settings). Sequencing depth and coverage were precisely determined using Mosdepth v0.3.2 [34]. Furthermore, we employed SAMtools [35] to convert SAM files to BAM files and subsequently sorted and indexed the BAM files and identified PCR duplications with Picard v2.21.4 (http://broadinstitute.github.io/picard).

### Prediction of ME fragments in the Sscrofa11.1 genome

We used the de-novo method to identify MEs of the Sscrofa11.1 genome using RepeatModeler (V1.0.11) [36], which employs clusters of repetitive sequences in the genome based on various methods and can automate runs of RECON (Multiple alignment clustering) and RepeatScout (Consensus seed clustering) for the pig genome. RepeatModeler analyzes the input genome sequence data, identifies duplicate sequences, and generates a set of duplicate sequence models. These models can be used for further genome annotation, research on genome structure and evolution, and identification of important genomic elements. We filtered the RepeatModeler annotations using the following criteria: 1) fragments smaller than 80 bp were removed; and 2) sequences of types such as “satellite”, “simple_repeat”, “low_complexity” and “unknown” were removed. The clean annotations were subsequently combined with public annotations from Dfam release 3.7 (https://dfam.org) to create the final version of the nonredundant ME annotation.

### Genotyping of MEVs in 747 samples using MEGAnE

Statistical genetics requires accurate genotyping of variant genes. To discover and accurately classify MEV genotypes from the genomes studied using short reads, we employed a new bioinformatics tool, the mobile element genotyping analysis environment (MEGAnE) [16]. This tool divides MEVs into mobile element absent (MEA) and mobile element insert (MEI) and has a low false-positive rate, a low missing rate, and high accuracy. Although there are some inherent limitations in the discovery of MEVs due to read length, the low false-positive rate and accurate genotyping offered by this tool allow us to query MEVs in short read data at previously impossible resolutions. We used MEGAnE to detect variation in 1,417 ME subfamilies across 747 samples, identifying a total of 147,993 polymorphic MEV loci. A MEV density map was constructed with the CMplot package in R (https://github.com/YinLiLin/CMplot). We also employed join-calling for the SNP/InDel genotypes of 747 samples using the PlatyPus pipeline [37].

### Allele spectrum analysis

We used PLINK v1.9 [38] to perform allele spectrum analysis on the population and breeds. We used a 5% threshold to divide allele frequencies into low and common MEVs. Breed-specific MEV analysis was also performed for 11 varieties with sample sizes greater than 9. We defined MEVs with allele frequencies greater than 30% in one variety and those with frequencies less than 5% in the other 10 varieties, which also overlapped with protein coding genes, as breed-specific MEVs.

### Enrichment analysis

In this study, we employed four active epigenetic markers as histone modifications: H3K4me1, H3K4me3, H3K27ac, and H3K36me3, along with an assay for transposase accessible chromatin with high-throughput sequencing (ATAC) intervals, which were retrieved from the Functional Annotation of Animal Genomes (FAANG) project [39]. Additionally, we downloaded 15 distinct chromatin states spanning 14 tissues from the project. To annotate the genome, we obtained gene location information from the Ensembl database (https://useast.ensembl.org/Sus_scrofa/Info/Index/). These data were downloaded and extracted for further analysis. Using the “merge” functionality in BEDtools [40], we grouped the overlapping peaks into consolidated regions. Next, we employed the “intersect” option in BEDtools to conduct an enrichment analysis on polymorphic MEVs, expression MEVs (eMEVs) and splicing MEVs (sMEVs).

### Population genetics and FST analysis

We conducted the following population genetic analysis on the MEV library constructed based on whole genome sequencing data from 747 individuals. We performed principal component analysis (PCA) using PLINK software. We used VCF2Dis (https://github.com/hewm2008/VCF2Dis) and FastMe [41] (http://www.atgc-montpellier.fr/fastme/) to build an Neighbor-Joining tree and visualize it through iTOL [42] (https://itol.embl.de/). To identify the divergent MEVs between different populations, we utilized VCFtools [43] to perform fixation index (F_ST_) analysis. This analysis was conducted between Asian domestic pigs and Asian wild pigs, as well as Asian pigs and European pigs. The top 1% of the F_ST_ values were defined as significantly differentiated MEVs. In addition, we downloaded expression data from muscle and adipose tissues from PigGTEx to perform differential gene expression analysis.

### Transcriptome quantification

For the NGS data of the 266 livers from F6, we first used Fastp for quality control. We used HISAT2 [44] and featureCounts software [45] to quantify gene expression in the 266 liver samples of F6 and Leafcutter [46] to leverage information from reads that spaned introns to quantify clusters of alternative spliced (AS) introns. Genes that were not expressed in any samples were removed, and the expression matrix was standardized using the package preprocessCore (https://bioconductor.org/packages/release/bioc/html/preprocessCore.html) in R.

### Detection of eMEVs and sMEVs in liver tissue

PEER (https://github.com/PMBio/peer) is a collection of Bayesian approaches used to uncover hidden determinants and their effects from gene expression profiles using factor analysis methods. Based on the gene expression matrix and percentage of spliced in (PSI) matrix, we obtained gene expression and PSI residues corrected by PEER and used them as covariates. Other covariates included the following: age, sex, weight, batch and the first three PCA scores for each sample. We identified MEVs as genotypes and used gene expression levels and PSI as phenotypes. We then performed standard expression eMEV and sMEV analysis using FastQTL [47]. We analyzed the data with 1,000 permutations and used Bonferroni correction to achieve the significance threshold.

### Bayesian fine-mapping analysis for eMEVs and sMEVs

We conducted fine-mapping analysis on 4,169 eMEVs and 6,914 sMEVs using FINEMAP [48]. For each gene, we extracted its eMEVs/sMEVs and the top 100 SNPs with the highest *P*-values to form a VCF file. Fine-mapping analysis was subsequently conducted according to the default parameters.

### SNP–MEVs reference haplotype construction and application

We constructed a SNP–MEV reference haplotype panel based on the mature pipeline that was previously established [16]. In brief, we kept all MEVs with call rates greater than 80%, and common SNPs with call rates greater than 95%, and then merged them into one VCF using BCFtools [49]. Next, we phased the haplotype reference panel using SHAPEIT5 [50]. Furthermore, the performance of the panel was evaluated using a leave-one-out (L1O) analysis. To show the application of this panel, we then conducted genotype imputation analysis for 5,457 samples from PigGTEx and 2,612 samples from a GWAS study [24] using Minimac3 [51] with default parameters. Specifically, we downloaded gene expression (TPM) matrix, alternative splicing excision level, and genotype files for 34 tissues from PigGTEx (https://piggtex.ipiginc.com/#/downloads). The genotype files of each tissue were filtered using PLINK: –maf 0.05 –geno 0.05. The filtered genotype files of the 34 tissues were subsequently imputed with Minimac3 based on our SNP–MEV reference panel. Finally, the eMEV and sMEV analyses for the 34 tissues were performed using FastQTL. We also downloaded 2,797 duroc pig genotype files from a previous study [24] and retained 2,612 individuals with complete phenotype records. PLINK was used to filter the genotypes, and the filtering parameters were as follows: –min-alleles 2 –max-alleles 2 –mac 10 –maf 0.02 –geno 0.05. The filtered genotype files for the 2,612 individuals were imputed with Minimac3 based on our SNP–MEV reference panel. We then used the mixed linear model in GEMMA [52] software to examine candidate MEVs associated with seven phenotypes, including total teat number (TTN), left teat number (LTN), right teat number (RTN), back fat thickness at 100 kg (BF, mm), loin muscle depth at 100 kg (LMD, mm), lean meat percentage at 100 kg (LMP, %), and time spent to eat per day (TPD, min).

## Results

### Construction of a mobile element variants database

**Fig. 1:**
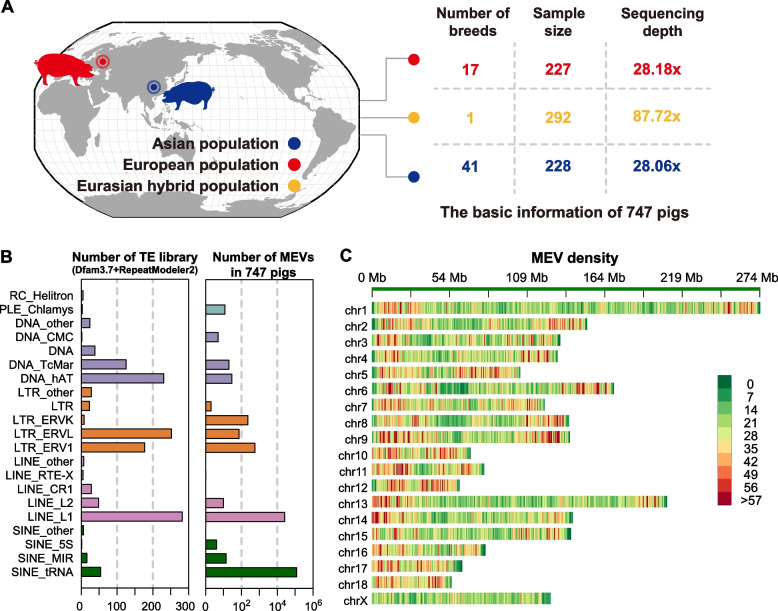
Distributions of MEVs in the genome of 747 pigs. **A** Overviews of whole genome resequencing of 747 individuals from three populations used in this study. Asian population (blue), European population (red), and Eurasian hybrid population (yellow). **B** Barplot showing the number of each ME subfamilies in Sus scrofa11.1 and the number of MEVs in 747 pigs. **C** The MEVs density distribution map shows the distribution of MEVs on 18 autosomes and X chromosome

A total of 747 high-depth porcine NGS datasets were generated in our laboratory and collected from two public databases (NCBI and CNGBdb). This dataset consists of paired-end reads generated on Illumina or BGI DNA nanoball (DNB) nanoarrays platforms, with a sequencing depth of at least 20× . The total volume of raw data reached 99.86 Tb in this study (Table S1). Our samples included major pig breeds representing two independent domestication centers, with 228 individuals from 41 breeds representing Asian pigs and 227 individuals from 17 breeds representing European pigs (Fig. 1A). To obtain a more comprehensive MEV landscape, we also applied an additional 292 hybrid F6 pigs generated from four Asian ancestors (Bama Xiang, Erhualian, Laiwu and Tibet pigs) and four European ancestors (Duroc, Yorkshire, Landrance and Hampshire) in our lab, with an average sequencing depth greater than 87× (Fig. 1A). Among the 747 samples, 380 individuals were sequenced at a high coverage depth by our laboratory, including the specifically designed hybrid F6 populations.

We applied MEGAnE to analyze the polymorphisms of MEVs in the 747 pigs and obtained a high-quality MEV atlas, which included 121,099 SINEs, 26,053 LINEs, 802 LTRs, and 39 DNA transposons (Fig. 1B). Compared with previous studies, we detected 54.18% (147,993 vs. 67,813) novel polymorphic MEVs [53]. Moreover, we found that there were certain positional characteristics in the distribution of MEVs, with a higher density of MEVs at both ends of the chromosome and a lower density in the middle (Fig. 1C). Compared with those of the other chromosomes, the MEV density on chromosome X was the lowest.

We used two strategies to evaluate the accuracy of the MEV genotypes. First, we assessed the consistency of MEVs detected in the NGS data of three pigs with SVs detected in the corresponding PacBio third-generation sequencing (TGS) data. Second, we measured the Mendelian genetic error rate of MEVs using 12 trio families. The results revealed that the accuracy of MEVs was greater than 85% (Fig. S1A), and the Mendelian average consistency of 12 trios was greater than 98.5% (Fig. S1B and Table S2), indicating the reliability of MEVs in our database.

### Distribution and function analysis of MEVs

**Fig. 2:**
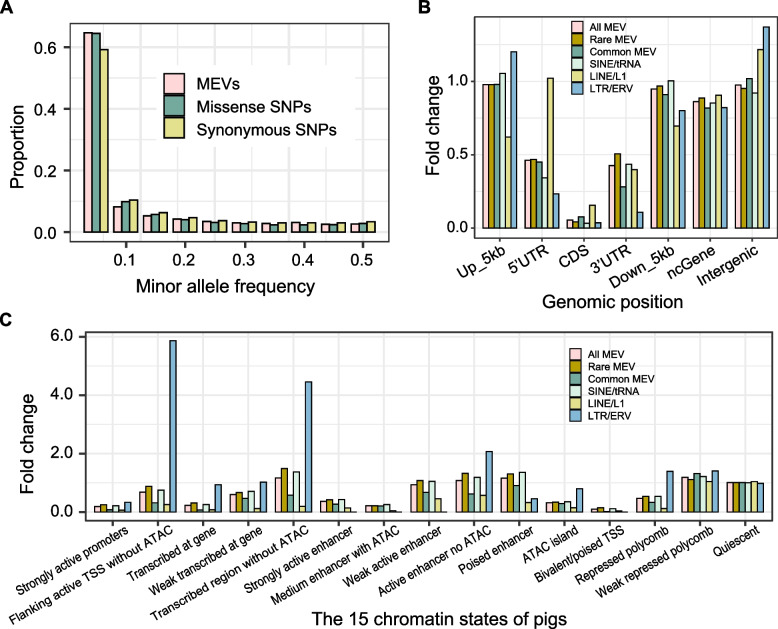
Enrichment analysis of MEVs in pig genome. **A** The proportion of MEVs and Missense SNPs and Synonymous SNPs. The *x*-axis represents the distribution of minor allele frequency (MAF) intervals, while the *y*-axis represents the percentage of different MEVs types within each interval. **B** The Fold Change of each MEV type at different genomic locations. Rare MEV (MAF < 5%), Common MEV (MAF > 5%). **C** The Fold Change of each MEV type at the 15 chromatin states of pigs

To fully characterize the features of MEVs, we first investigated the allele spectrum of MEVs in detail. We found that the minor allele frequency of MEVs was similar to that of SNPs. More than 60% of MEVs had minor allele frequencies less than 5%, a proportion similar to that of missense SNPs (Fig. 2A). MEVs were exhausted more than synonymous and missense SNPs, especially in the lower frequency groups (< 10% and < 15%). These results indicate that MEVs suffer from strong negative selection effects. With respect to the length distribution, the major peak of the SINEs were approximately 300 bp, that of LTRs were approximately 1 kb, and that of LINEs were relatively uniform, which may have been caused by an insufficient ability to detect large MEVs from short reads (Fig. S2A).

To explore the potential functions of MEVs, we also downloaded the gene annotations (version 110) of Sscrofa11.1 from the Ensembl database. We then extracted the coding sequence (CDS) regions, UTRs, noncoding gene (ncGene) regions, intergenic regions, and the 5 kb regions upstream and downstream of genes (Fig. 2B). Analysis of the MEVs in these regions revealed that the MEVs tended to be more enriched in the intergenic and ncGene regions, with MEVs in the CDS regions being the sparsest. Mutations in CDS regions are generally harmful and can negatively impact the survival of organisms, making them unlikely to be retained. We also found that rare MEVs were more enriched in 3′-UTRs than common mutations, possibly because the presence of MEVs in 3′-UTRs contributes to environmental responses and RNA stability [54]. Overall, the enrichment of MEVs in the 5 kb upstream regions of the genes was greater than that in the 5′-UTRs, possibly because MEVs may function as cryptic transcription start sites (TSSs) to increase transcription, as previously demonstrated [54]. Furthermore, we observed that LINE/L1 elements were significantly more enriched in the CDS and 5′-UTR regions than other types of transposed elements, which may be related to their active properties [55].

The FAANG project defines chromatin in 15 states based on data from 14 types of pig tissues and multi-omics data [39]. We examined the overlap and enrichment of MEVs with chromatin states, and the results revealed that MEVs were present in all 15 chromatin state regions, but did not exhibit enrichment features overall (Fig. 2C). Interestingly, MEVs were depleted in functional regions including active promoters and enhancers, which are transcribed at genes and TSS regions. These results further suggest that MEVs are subject to strong negative selection pressure. In contrast, we found that LTR/ERV elements were significantly enriched in “flanking active TSS regions without ATAC signals” and in “transcription regions without ATAC signals”. The reasons for these enrichments remain to be explored. In summary, these results suggest that MEVs are selected against in functional regions and found in regions without ATAC signals.

### Contributions of MEVs to domestication and breed differentiation

**Fig. 3:**
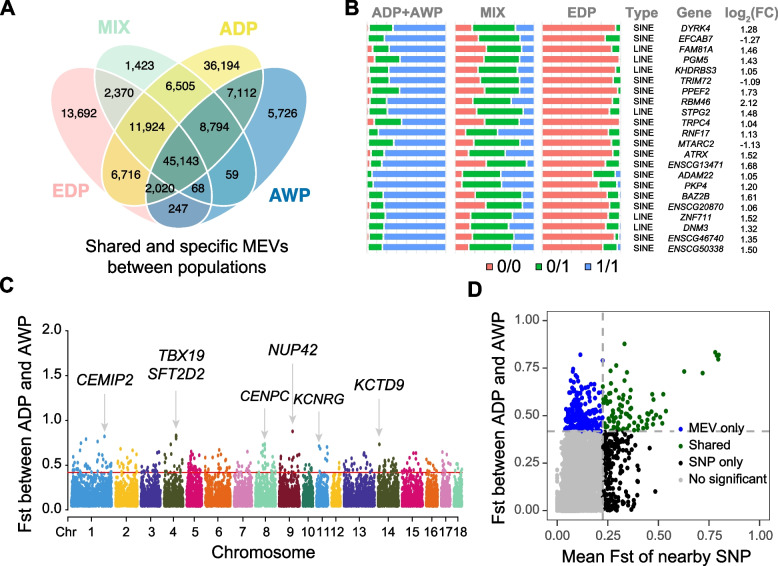
Pairwise comparisons of F_ST_. **A** Venn diagram shows the shared/specific MEVs between different groups. European domestic pigs (EDP), Eurasian hybrid population (MIX), Asian domestic pigs (ADP) and Asian wild pigs (AWP). **B** The genotypic distribution of 22 MEVs in three populations was displayed, with at least one nearby gene showing significant differential expression in muscle tissue between Asian and European pigs. **C** The F_ST_ values between Asian domestic pigs and Asian wild pigs. The top 1% values are defined as significantly differentiated variants. **D** Comparison the F_ST_ of MEVs and their nearby SNPs. MEV only (blue), SNP only (green), SNP only (black), No significant (gray)

To further explore the MEV characteristics of global pig populations, we conducted a population genetic analysis on high-quality polymorphic MEVs from 747 individuals representing all 59 subspecies of the four major pig populations. The principal component analysis and phylogenetic tree analysis results revealed that the four populations of Asian domestic pigs, Asian wild pigs, European pigs, and Eurasian hybrid pigs could be distinguished clearly based on genome-wide MEVs (Fig. S3). This finding is consistent with the results generated by genetic markers such as SNPs and short tandem repeats (STRs). Furthermore, we detected many shared and specific MEV alleles across the four populations, with the number of specific MEV alleles in Asian pigs being significantly greater than that in European domestic pigs (Fig. 3A). Asian domestic pigs and European domestic pigs had the highest number of population-specific MEVs, with 36,194 and 13,692, respectively, indicating that there are still many differences between European and Asian pigs. In contrast, the numbers of population-specific MEVs of wild pigs and Eurasian hybrid pigs were relatively small, at 5,726 and 1,423, respectively. The lower number of specific MEVs in wild pigs may be due to the presence of fewer individual populations, whereas Eurasian hybrid pigs likely have ancestors from multiple breeds of Asian and European pigs, resulting in the lowest number of specific sites (Fig. 3A). Additionally, the average number of polymorphic MEV loci in Asian pigs was greater than that in European pigs, indicating that Asian pigs possess the highest genetic diversity. Notably, the genetic diversity of Eurasian hybrid pigs (F6 population) is also very high and these pigs can be used as excellent materials for studying MEVs in the future (Fig. S2B). Next, we focused on MEVs associated with domestication and differentiation. By F_ST_ analysis, we found 514 significantly different MEVs between Asian and European pigs. Among these, 294 MEVs within a ±10 kb range surrounded 287 protein-coding genes (Table S3). Functional enrichment analysis revealed that these genes were enriched in the mitogen-activated protein kinase (MAPK) family signaling cascades and lipid translocation pathways (Fig. S4A). We further examined the differential expression of these genes in Asian and European pig muscle tissue and identified 22 significant differentially expressed genes (Fig. 3B). For example, the *ATRX* gene was differentially expressed in muscle tissue between Asian and European populations and contained a SINE element that was also significantly different between the two populations. Previous studies have demonstrated the importance of *ATRX* in muscle development [56, 57]. We also identified 530 candidate MEVs that significantly differed between Asian domestic pigs and Asian wild boars, with 291 genes around these loci enriched in the regulation of reproductive processes, nervous system development, and immune system process pathways (Fig. S4B and Table S4). The *TBX19* and *NUP42* genes have also been reported in previous studies based on SNP/STR/SV analyses (Fig. 3C). Notably, we found that 74.34% of domestication-related MEVs could not be reproduced by SNP haplotypes (Fig. 3D). These results demonstrate that MEVs add significant value to population genetic analysis.

Breed-specific MEVs are among the potential factors contributing to the development of different pig breeds. Therefore, we selected 11 varieties with individual quantities greater than or equal to 9 for differential analysis. We calculated the allele frequencies of MEVs for each breed and selected MEVs that overlapped with protein-coding genes. We subsequently conducted a differential analysis and defined MEVs with allele frequencies greater than 0.3 in one breed and less than 0.05 in the other pigs as breed-specific MEVs. We identified 584 breed-specific MEVs that overlapped with genes in all 11 breeds, some of which were related to pig size and growth and development (Fig. S5). Among them, 327 MEVs within a ±5 kb range surrounded 337 protein-coding genes, which may be one of the potential reasons for the formation of different pig breeds and 257 MEVs that were not within a ±5 kb range surrounded protein-coding genes (Table S5). For example, the *TCF21* gene in Duroc, which was influenced by a breed-specific MEV, was identified as a candidate gene for reproductive traits in a GWAS of 1,075 boars [58]. We found that, in Bama Xiang pigs, the *PCM1* gene influenced by breed-specific MEV may be a candidate gene affecting pig body size [59]. In the wild boar population, we also discovered a breed-specific MEV within the *FAM78B* gene that affects immunity [60].

### MEVs regulate gene expression and alternative splicing in liver tissue

**Fig. 4:**
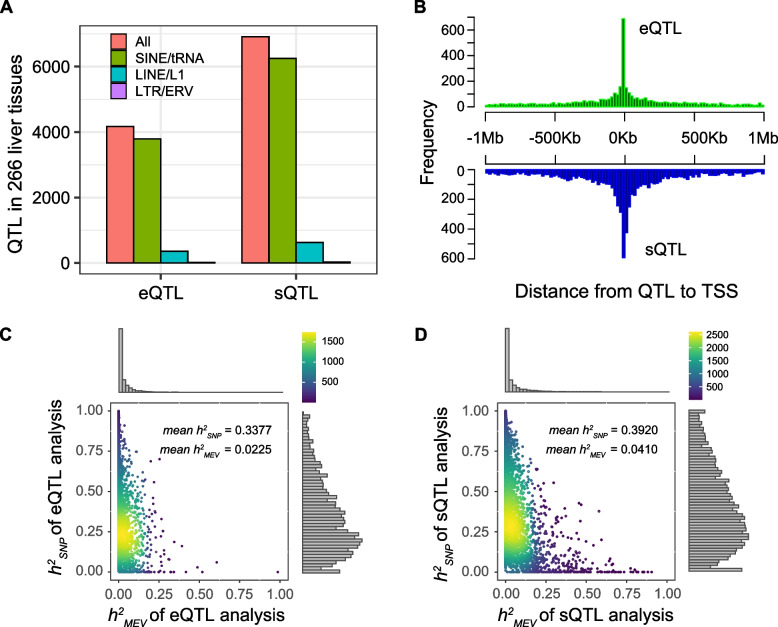
eMEVs and sMEVs analysis. **A** The number of eMEVs and sMEVs in 266 liver tissues. **B** The frequency of distance from eMEVs and sMEVs to the TSS. **C** and **D** Heat scatter plots showing the heritability of each QTL apportioned to the most significant MEVs in the cis window (*x*-axis) and the additive effect from the top 1,000 most significant SNP in the cis window (*y*-axis) for eMEVs and sMEVs mapping analysis

To explore the potential functional role of MEVs at the transcriptome level, we evaluated the potential regulatory effects of MEVs on gene expression and alternative splicing. Based on a panel of liver RNA sequencing data from a population of 266 heterogeneous F6, we performed standard expression quantitative trait locus (eQTL) and splicing quantitative trait locus (sQTL) analyses using FastQTL, and a total of 4,169 eMEVs and 6,914 sMEVs were identified in the 266 samples from the F6 population (Fig. 4A, Tables S6 and S7). The results revealed that SINEs/tRNAs were the main components of both eMEVs and sMEVs, followed by LINEs/ERVs. In addition, eMEVs and sMEVs were both close to the TSSs (Fig. 4B). Heritability partitioning revealed that the eMEVs accounted for 6.24% of the *cis*-heritability of gene expression, and sMEVs accounted for 9.47% of the *cis*-heritability of splicing. Some MEVs explained more heritability than all significant SNPs in *cis*-regulatory regions did, indicating that they are more likely to be causal variations (Fig. 4C and D).

Next, we examined the enrichment of eMEVs and sMEVs in different functional regions (Fig. S6). We found that the distributions of eMEVs and sMEVs in the genome were generally similar and that both were significantly enriched 5 kb upstream and 5 kb downstream of genes. An exception was L1-related QTLs, which were enriched in the CDS and 5’-UTR regions. In addition, our evidence did not support eMEV and sMEV enrichment in epigenetic regions such as ATAC, H3K27ac, H3K4me1, and H3K4me3.

**Fig. 5:**
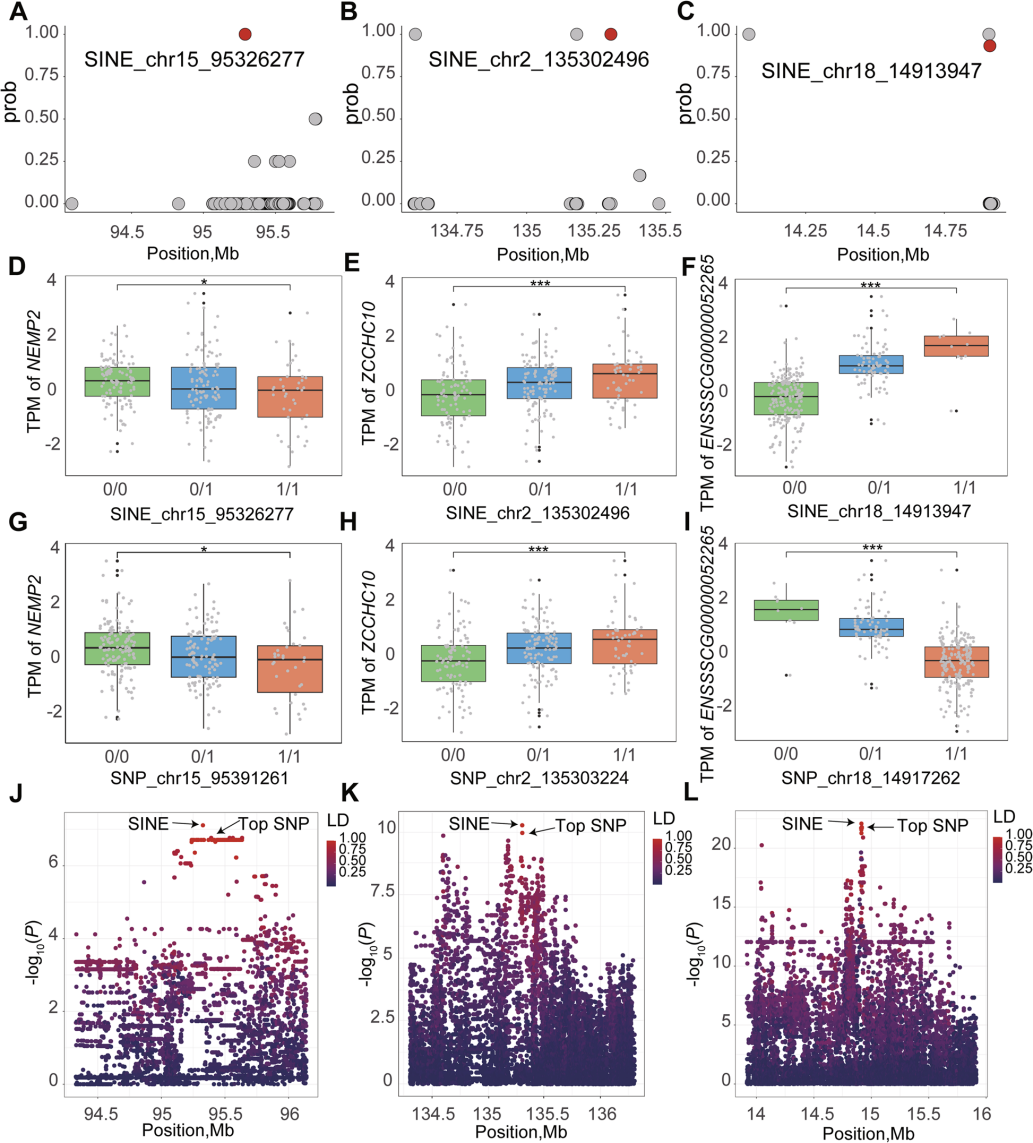
Examples of candidate eMEVs. **A**–**C** Fine-mapping analysis of the posterior probabilities of the MEV with the highest *P*-value and the nearby 100 SNPs with the highest *P*-values for eQTL. The red dots represent eMEVs, which are *MEMP2*-SINE_chr15_95326277 (**A**), *ZCCHC10*-SINE_chr2_135302496 (**B**) and *ENSSSCG00000052265*-SINE_chr18_14913947 (**C**), respectively. The gray dots represent SNPs. **D–F** The effect of different MEV genotypes on the expression of gene. *MEMP2*-SINE_chr15_95326277 (**D**), *ZCCHC10*-SINE_chr2_135302496 (**E**) and *ENSSSCG00000052265*-SINE_chr18_14913947 (**F**). **G**–**I** The effect of top SNP genotypes on the expression of gene. *MEMP2*-SINE_chr15_95391261 (**G**), *ZCCHC10*-SINE_chr2_135303224 (**H**) and *ENSSSCG00000052265*-SINE_chr18_14917262 (**I**). **J–L** Local visualization of the gene association with eMEV and eSNP. The vertical axis represents the *P*-value, and the color represents the LD degree. *MEMP2* (**J**), *ZCCHC10* (**K**), *ENSSSCG00000052265* (**L**)

We conducted standard Bayesian fine mapping analysis on 4,169 eMEVs identified in the 266 livers from F6 population, and identified a total of 531 eMEVs with posterior probabilities greater than 0.5 (Table S8). The corresponding genes were mostly enriched mostly in metabolic pathways of the liver, such as the cellular catabolic process, purification containing compound metabolic process, and small molecular catabolic process, suggesting the regulatory effect of MEV on liver gene expression and function (Fig. S7). For these 531 eMEVs, we further identified 4 eMEVs that were more significant than nearby SNPs. For example, SINE_chr15_95326277 (*P* = 7.75581e-08), SINE_chr2_135302496 (*P* = 5.62539e-11), and SINE_chr18_14913947 (*P* = 8.30468e-23) were the top eMEVs of *MEMP2*, *ZCCHC10*, and *ENSSSCG00000052265*, respectively. Their posterior probabilities were all above 0.9 (Fig. 5A–C). The expression profiles of these three genes significantly differed according to their corresponding MEV and top SNP genotypes (Fig. 5D–I). Additionally, MEVs and adjacent SNPs showed extensive linkage disequilibrium (Fig. 5K–L). We also conducted standard Bayesian fine mapping analysis on 6,914 sMEVs and found a total of 720 sMEVs with posterior probabilities greater than 0.5 in the 266 livers from F6 population (Table S9). Overall, we believe that the abovementioned MEVs are more likely to be causal mutations affecting gene expression or AS and function in liver of pig. Taken together, these results also demonstrate the important role of MEV in regulating gene expression.

### Construction and application of SNP–MEV reference haplotypes

**Fig. 6:**
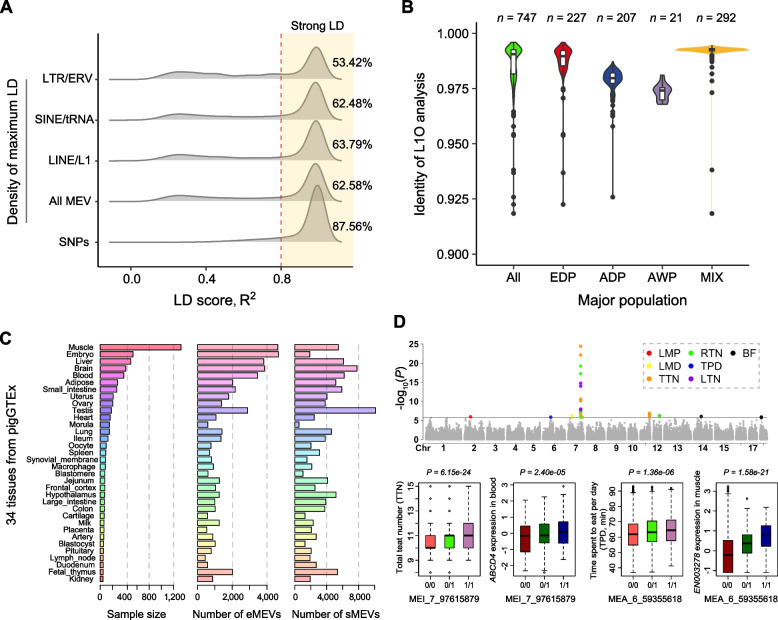
SNP–MEV haplotype reference panel and its application. **A** Density of the maximum LD between SNPs and different types of variants. **B** L1O analysis for different groups. EDP (European Domestic Pig), ADP (Asian Domestic Pig), AWP (Asian Wild Pig), MIX (Eurasian hybrid population). **C** The sample size, number of eMEVs, and number of sMEVs in 34 tissues of PigGTEx. **D** ﻿The manhattan plots of MEV-GWASs for seven economic phenotypes. The *x*-axis shows chromosomes. The *y*-axis shows significant levels of association. The box plot distribution shows the relationship between MEVs genotypes and transcription or phenotype

Previous studies have often considered only the effects of SNPs, whereas the contribution of MEVs to complex traits has often been overlooked. Therefore, we calculated the linkage disequilibrium (LD) of all SNPs within the ±100 kb region and the LD between SNPs and different MEVs. We observed that 87.56% of the SNP–SNP pairs exhibited strong LD (R^2^ > 0.8). Overall, 62.58% of the MEV–SNP pairs reached strong LD, and LTR/ERVs presented the lowest degree of linkage with SNPs of different MEV types (Fig. 6A). These results indicate that some MEVs may have their own genetic effects independent of SNPs. Previous research has also indicated that incorporating a haplotype reference panel has the potential to increase the resolution and statistical power of GWASs [61, 62]. We constructed a SNP–MEVs haplotype reference panel by integrating 74,630 high-quality MEVs and 22,510,073 common SNPs. Simultaneously, we conducted L1O analysis to verify the accuracy of genotype imputation using this reference panel. The results demonstrated that the concordance rate between the imputed MEV genotypes and actual genotypes exceeded 97.5% across different populations (Fig. 6B). These results inspire us to explore unknown MEV-related regulatory effects from publicly available data using genotype imputation analysis.

We applied our reference panel to the PigGTEx project data and performed standard eQTL and sQTL analyses with the imputed MEVs. This led to the identification of hundreds of eMEVs and sMEVs across 34 tissues. In detail, we identified 20,916 unique eMEVs associated with 18,424 genes in at least one tissue, as well as 28,276 unique sMEVs associated with 69,507 splicing events. And we observed a significant positive correlation between the number of eMEVs/sMEVs and sample size, with the exception of testis tissue and fetal thymus, where very high levels of eMEVs/sMEVs were identified regardless of sample size (Fig. 6C). Further statistical analysis found that the majority of eMEVs and sMEVs were tissue-type specific, with an average of 21.13% eMEVs and 17.92% sMEVs shared between tissues (Fig. S8A and B). Moreover, on average, approximately 267 lead expression variants and 670 lead splicing variants in each tissue were MEVs compared to SNPs, respectively (Fig S8C and D). Together, these results highlight the importance of integrating our reference panels with pigGTEx resources. Additionally, we applied our reference panel to a GWAS project involving 2,661 commercial pigs and performed standard MEV-GWAS analyses with the imputed MEVs. As a result, we identified a candidate MEV associated with total teat number (MEI_7_97615879, *P* = 6.15e-24), which was also associated with *ABCD4* expression (MEI_7_97615879, *P* = 2.40e-05) in blood in PigGTEx. Notably, a recent study revealed that *ABCD4* is a key candidate gene for regulating mammary gland development and lactation during pregnancy [19]. Another case is MEA_6_59355618, which impacts time spent to eat per day traits (*P* = 1.36e-06) in commercial pigs and significantly affects *ENSSSCG0000003278* expression in muscle in PigGTEx (Fig. 6D). These results suggest that our haplotype reference panel has substantial potential for various applications.

## Discussion

MEVs have become important genetic markers for studying the genomes and breeding of different agricultural animals [63–65]. However, exploration of MEV resources at the population level of the pig genome is still scarce, especially compared with the available resources for single-nucleotide variants (SNVs) and SVs [25, 66]. Various types of MEVs are widely present in the pig genome but are not randomly distributed. SINEs and LINEs are more abundant than LTR and DNA transposons. Our research revealed that SINE-tRNA was the most abundant type, which is consistent with the finding of previous studies. Notably, SINEs have previously been reported to contribute many polymorphisms to the pig genome [67]. Next is LINE1, which is also one of the most active transposons in humans. Recently, several excellent articles have highlighted the causality of MEVs, but most of them have focused solely on a limited number of loci [68, 69]. Additionally, some studies have used an insufficient number of pig breeds or sample sizes [53, 70], and few studies have constructed haplotype reference panels based on MEVs to improve the resolution and statistical power of GWASs [61, 62].

In this study, we constructed a comprehensive MEV resource and SNP–MEV reference haplotype panel using a total of 747 individuals from 58 breeds from Asia and Europe, as well as a hybrid variety. Compared with previous studies, 54% of the MEVs were newly discovered in our dataset. A comprehensive database that encompasses all MEVs across the pig genome can provide detailed and accurate genetic background knowledge for researchers. This database offers high-resolution information on MEV loci, enabling precise localization and analysis of the impact of MEVs on gene function and genome structure. By including MEVs from various pig breeds and populations, the database aims to analyze and understand the genetic diversity and population structure of domestic pigs. Furthermore, by integrating MEVs with gene expression and phenotypic data, the database provides rich functional annotation information and reveals the specific impacts of MEVs on gene expression and biological functions.

In the past 20 years, haplotype reference panels have received increasing attention. Renowned large-scale projects such as the International HapMap Project [71] and the 1000 Genomes Project [72] have produced high-quality and rich reference haplotype panels. These resources are mainly used for genotype filling of other data to improve the accuracy and richness of genotypes. With the enrichment of reference haplotype panels, the accuracy of genotype imputation and subsequent accuracy for GWASs will increase. However, these large-scale projects are all based on human samples. In recent years, there have been reports on reference haplotype reference panels for pigs, but they are all constructed based on SNPs [25]. Owing to the important role of MEVs and the difficulties in their detection, it is necessary to construct a rich and accurate MEV haplotype reference panel. In this study, we successfully constructed the most comprehensive SNP–MEV reference haplotype panel currently available. We also present a case study demonstrating its potential application in the genetic analysis of complex traits. Using this reference panel, we can accurately impute missing MEV genotypes in the target population, thereby improving the accuracy and efficiency of GWAS analysis. In summary, this study provides valuable genetic resources for subsequent breeding and genetic improvement work. We also look forward to further optimizing and improving the richness and accuracy of this reference panel in future research, with the aim of discovering more genetic variations related to complex traits.

Multiple lines of evidence from our manuscript suggest that MEVs suffer from strong negative selection pressures. This phenomenon is similar to those of other genetic variants, such as short tandem repeats and structural variants, in the pig genome [73]. Through systematic analysis of WGS data from multiple varieties, we identified a group of MEVs with significant differences between populations and revealed their potential functions in driving variety formation and population adaptation. These differences indicate that MEVs play an important role in population differentiation, possibly as a result of the combined effects of environmental selection pressure and genetic drift. MEVs can also supplement some of the signals associated with evolution, domestication, and selection that are not captured by SNP markers. In fact, the majority of *cis*-regulatory elements newly evolved during primate evolution are directly derived from MEs [74, 75]. Recent research has demonstrated that PRE-1, an active subfamily of MEs, can mediate structural differences between Asian and European pigs through introgression mechanisms [76]. In addition, Chen et al. systematically analyzed SINE maps in the pig genome, highlighting the importance of polymorphic SINE markers in genetic diversity and population differentiation [67, 77].

In this study, we suggest potential functions and regulatory mechanisms of MEVs in the pig genome. We successfully identified 4,169 eMEVs and 6,914 sMEVs that significantly affect liver gene expression in a large population. Specifically, eMEVs explained 6.24% of the variation in *cis*-heritability, whereas sMEVs explained 9.47% of the variation in *cis*-heritability. This finding recovered the heritability overlooked in SNP-related research. MEs affect not only gene expression but also the variable splicing of mRNAs and the selective use of reference or secondary transcripts, which has been confirmed in humans, mice and Drosophila [54, 78–80]. MEs have the ability to incorporate functional promoters into novel genomic positions, thus resulting in the formation of new host gene transcripts [81, 82]. These alternative promoters are regulated by tissue-specific and developmental stage specific expression [81, 83, 84]. Overall, MEs can regulate gene expression or further drive phenotype formation, but this process is very complex. It may involve the regulation of transcription factor binding sites (TFBSs), promoters, enhancers, modifications of three-dimensional chromatin architecture, and overall effects of ME silencing mechanisms [85, 86].

In pigs, some studies have demonstrated that ME insertion is a causal mutation of important economic traits [15]. Although this study identified some candidate MEVs associated with traits such as the total number of teats and feeding time, it is important to note that these associations could be influenced by hitchhiking effects linked to nearby SNPs [87]. Therefore, additional analysis methods and validation experiments should be integrated to identify causal MEVs for complex traits in future research.

The abundance and potential perniciousness of endogenous retroviruses (ERVs) in the pig genome are important concerns in the fields of genomic research and xenotransplantation. PERVs are present in the genomes of all pigs and have the ability to generate viral particles that can infect human cells, thus posing a special risk in xenotransplantation. The quantity and distribution of PERVs in the pig genome are central questions related to their biology and potential harm. Our dataset contains 802 LTR/ERV variant loci, and these MEVs show high polymorphism across individuals and breeds, although they are not directly annotated as PERVs. In fact, the number of copies of PERVs varies significantly across pig breeds and individuals, ranging from a few to hundreds [88]. There are at least 36 complete PERVs and 23 incomplete PERVs in the pig reference genome Sscrofa11.1, and the copy number in European pigs is significantly greater than that in Asian pigs, with the lowest copy number in Bama Xiang pigs [89]. Moreover, the number of PERVs in different organs also varies and increases with increasing age, suggesting that PERVs are active in living pigs and are able to replicate and integrate into new genomic locations [90].

Notably, LTR/ERV elements were significantly enriched in “flanking active TSS regions without ATAC signals” and in “transcription regions without ATAC signals” in our dataset. This pattern suggests that these retroviral elements preferentially integrate into or become silenced in genomic regions with reduced chromatin accessibility, potentially serving as a natural regulatory mechanism to mitigate their disruptive impact on the host genome. Because retroviruses can cause tumors and immune deficiencies, PERVs pose significant safety concerns in xenotransplantation. Although there is currently no evidence of transmission of PERVs to humans in clinical and animal xenotransplantation trials, their potential pathogenicity and ability to spread across species still require further study and vigilance [91]. The evolution and recombination of PERVs have further increased their potential for harm. PERVs are spread across species by precursors of retroviruses from different animals and have evolved further in the pig genome to form different long terminal repeats (LTRs) and recombination events, which could have important effects on the transmissibility and pathogenicity of PERVs [90, 92]. Therefore, future studies need to continue to focus on the abundance, activity, chromatin status, and their possible pathogenic pathways of PERVs to ensure the safety and efficacy of xenotransplantation.

There are still several limitations in our research. For example, we did not provide results on the possible impact of ME copy number on gene expression, which may be important in the evolutionary process of pig breed formation. But our analysis is primarily based on existing short-read data. Because of the highly repetitive nature of transposable element sequences and their evolutionary relatedness among ME families, mapping short reads originating from ME is a real challenge [82, 93]. The software MEGAnE [16] we used, which is a better ME genotyping software than MELT [94], are well in determining the presence or absence of ME fragment, but it is difficult to accurately estimate the specific number of copies. Therefore, we only analyzed the effects of the MEI or MEA of MEs on gene expression and phenotype. In addition, our research focused mainly on the establishment of reference resources for MEVs and the associations between MEVs and complex traits in pigs. Therefore, the sample size needs to be expanded in terms of individual and breed numbers. In addition, more advanced omics data are needed to further improve our MEV resources. Finally, owing to technical and cost limitations, there is a lack of confident experimental results demonstrating the key functions of MEVs, such as their participation in breed formation. Notably, the existing reference genomes are poorly assembled in centromere and repeat regions, which may affect the identification and genetic analysis of MEVs. In the future, large-scale multibreed long-read sequencing data based on telomere-to-telomere (T2T) reference genomes, combined with multi-omics data, will be conducive to the in-depth analysis of the important biological functions of MEVs.

## Conclusion

In summary, in this study, the most comprehensive ME mutation resource library and SNP–MEV reference haplotype panel were constructed using an abundant population and sample set. The ME mutation resource library reveals the important roles of these mutations in population differentiation and breed formation, providing new insights into genetic diversity and evolutionary mechanisms. eMEV and sMEV analyses demonstrated the important relationship between MEVs and gene expression and retrieve previously overlooked heritability in SNP-related research. The construction and application of the SNP–MEV reference haplotype panel offer a high-quality genetic resource that surpasses what SNPs alone can provide.

## Supplementary Information

Additional file 1: Fig. S1. Assessment of MEVs genotyping accuracy. A) Concordance ratio of MEVs detected by Next-generation sequencing (NGS) and Third-generation sequencing (TGS) data for the three individuals. B) Mendelian genetic ratio of 12 trio families. Fig. S2. Characterization of MEVs in 747 pigs. A) The length distribution of four different types of MEVs. B) Cumulative distribution of MEVs, as the sample size increases in each group, the total number of MEV counts increases accordingly. And the MEV counts of each individual in each group, sorted in ascending order within the group. Fig. S3. Population genetic analysis using MEVs. A) Principal component analysis of all individuals. Different colors represent different populations. B) Neighbor-joining phylogenetic tree of pig breeds based on MEVs, with varieties represented by different colors consistent with the PCA diagram. The scale bar represents proportional to similarity. Fig. S4. Gene function enrichment analysis. A) Functional enrichment of 294 differential genes between Asian and European pigs. B) Functional enrichment of 291 differential genes between Asian domestic pigs and Asian wild pigs. Fig. S5. Breed-specific MEVs analysis. Heatmap showing breed specific MEVs that overlap with genes in all 11 breeds, and bar plot above heatmap represents number of breed-specific MEVs. Fig. S6. Distribution characteristics of eMEVs and sMEVs. A) The fold change of eMEVs at different genomic locations. B) The fold change of sMEVs at different genomic locations. C) The fold change of eMEVs at the four active epigenetic markers in liver tissue of pigs. D) The fold change of sMEVs at the four active epigenetic markers in liver tissue of pigs. Fig. S7. Gene enrichment with a posterior probability greater than 0.5. Functional enrichment of 531 genes with a posterior probability greater than 0.5. Fig. S8. eQTL and sQTL analysis of 34 tissues from pigGTEx data based on imputed MEVs. A) The number of MEV-eQTLs that are shared or specific between tissues. B) The number of MEV-sQTLs that are shared or specific between tissues. C) The MEV-eQTLs which lead expression variants are SNPs or MEVs. D) The MEV-sQTLs which lead splicing variants are SNPs or MEVs.

Additional file 2: Table S1. The basic statistic information of 747 samples. Table S2. Mendelian genetic ratio of 12 trios families. Table S3. The divergent MEVs between European pigs and Asian pigs. Table S4. The divergent MEVs between Asian domestic pigs and Asian wild pigs. Table S5. The breed-specific MEVs. Table S6. A total of 4169 significant eMEVs-Gene pairs. Table S7. A total of 6,914 significant sMEVs-PSI pairs. Table S8. Genes with a posterior probability greater than 0.5 for eMEVs. Table S9. Posterior probability greater than 0.5 for sMEVs.

## Data Availability

Scripts and supplemental datasets used in this study are available from: https://github.com/jxlabWzZ/Sus_MEVs_WGS. The 380 WGS data generated by our laboratory has been uploaded to the public database previously, and the accession number is listed in Supplemental Table 1. The PigGTEx data used for the analyses described in this manuscript were obtained from the PigGTEx-Portal (01/04/2024, http://piggtex.farmgtex.org/). The mobile element variant database and SNP–MEVs reference haplotype panel generated in this study have been deposited in the National Genomics Data Center database under accession code GVM000853 (https://ngdc.cncb.ac.cn/gvm/getProjectDetail?project=GVM000853).

## Abbreviations

ADP: Asian domestic pigs
AS: Alternative spliced
ATAC: Assay for transposase accessible chromatin with high-throughput sequencing
AWP: Asian wild pigs
BF: Back fat thickness at 100 kg
CDS: Coding sequence
CNV: Copy number variant
DNB: BGI DNA nanoball
eMEV: Expression MEV
eQTL: Expression quantitative trait locus
EDP: European domestic pigs
ERV: Endogenous retrovirus
F6: Sixth generation
FAANG: Functional annotation of animal genomes
F_ST_: The fixation index
GHR: Growth hormone receptor
GWAS: Genome-wide association study
HPC: High-performance computing centre
LMP: Lean meat percentage at 100 kg
L1O: Leave-one-out
LTN: Left teat number
LD: Linkage disequilibrium
LMD: Loin muscle depth at 100 kg
L1, LINE1: Long interspersed nuclear element 1
LINE: Long interspersed nuclear element
LMP: Lean meat percentage at 100 kg
LTR: Long terminal repeats
MAF: Minor allele frequency
MAPK: Mitogen-activated protein kinase
MEV: Mobile element variant
MEA: Mobile element absent
MEGAnE: Mobile element genotyping analysis environment
MEI: Mobile element insert
ME: Mobile element
MEV: Mobile element variant
MIX: Eurasian hybrid population
NGS: Next-generation sequencing
ncGene: Noncoding genes
PSI: Percent of spliced in
PigGTEx: Pig genotype-tissue expression
PERV: Porcine endogenous retroviruses
PCA: Principal component analysis
RTN: Right teat number
SINE: Short interspersed nuclear elements
sMEV: Splicing MEV
sQTL: Splicing quantitative trait locus
STR: Short tandem repeat
SNP: Single-nucleotide polymorphism
SNV: Single-nucleotide variant
Sscrofa11.1: *Sus scrofa* reference genome version 11.1
TGS: Third-generation sequencing
TPD: Time spent to eat per day
TTN: Total teat number
TFBS: Transcription factor binding site
TSS: Transcription start site
TE: Transposable element
T2T: Telomere-to-telomere
WGS: Whole genome sequencing

## References

[1] Ma J, Yang J, Zhou L, Ren J, Liu X, Zhang H, et al. A splice mutation in the *PHKG1* gene causes high glycogen content and low meat quality in pig skeletal muscle. PLoS Genet. 2014;10(10):e1004710. 25340394 10.1371/journal.pgen.1004710 PMC4207639

[2] Rubin CJ, Megens HJ, Martinez Barrio A, Maqbool K, Sayyab S, Schwochow D, et al. Strong signatures of selection in the domestic pig genome. Proc Natl Acad Sci U S A. 2012;109(48):19529–36. 23151514 10.1073/pnas.1217149109 PMC3511700

[3] Wells JN, Feschotte C. A field guide to eukaryotic transposable elements. Annu Rev Genet. 2020;54:539–61. 32955944 10.1146/annurev-genet-040620-022145 PMC8293684

[4] Griffith BP, Goerlich CE, Singh AK, Rothblatt M, Lau CL, Shah A, et al. Genetically modified porcine-to-human cardiac xenotransplantation. N Engl J Med. 2022;387(1):35–44. 35731912 10.1056/NEJMoa2201422 PMC10361070

[5] Montgomery RA, Stern JM, Lonze BE, Tatapudi VS, Mangiola M, Wu M, et al. Results of two cases of pig-to-human kidney xenotransplantation. N Engl J Med. 2022;386(20):1889–98. 35584156 10.1056/NEJMoa2120238

[6] Denner J, Tönjes RR. Infection barriers to successful xenotransplantation focusing on porcine endogenous retroviruses. Clin Microbiol Rev. 2012;25(2):318–43. 22491774 10.1128/CMR.05011-11 PMC3346299

[7] Sykes M, Sachs DH. Transplanting organs from pigs to humans. Sci Immunol. 2019;4(41):eaau6298. 31676497 10.1126/sciimmunol.aau6298 PMC7293579

[8] Mc CB. The origin and behavior of mutable loci in maize. Proc Natl Acad Sci U S A. 1950;36(6):344–55. 15430309 10.1073/pnas.36.6.344 PMC1063197

[9] Tang Y, Ma X, Zhao S, Xue W, Zheng X, Sun H, et al. Identification of an active miniature inverted-repeat transposable element *mJing* in rice. Plant J. 2019;98(4):639–53. 30689248 10.1111/tpj.14260 PMC6850418

[10] Liu Z, Zhao H, Yan Y, Wei MX, Zheng YC, Yue EK, et al. Extensively current activity of transposable elements in natural rice accessions revealed by singleton insertions. Front Plant Sci. 2021;12:745526. 34650583 10.3389/fpls.2021.745526 PMC8505701

[11] Diehl AG, Ouyang N, Boyle AP. Transposable elements contribute to cell and species-specific chromatin looping and gene regulation in mammalian genomes. Nat Commun. 2020;11:1796. 32286261 10.1038/s41467-020-15520-5 PMC7156512

[12] Roller M, Stamper E, Villar D, Izuogu O, Martin F, Redmond AM, et al. Line retrotransposons characterize mammalian tissue-specific and evolutionarily dynamic regulatory regions. Genome Biol. 2021;22(1):62. 33602314 10.1186/s13059-021-02260-y PMC7890895

[13] Casanova M, Moscatelli M, Chauvière L, Huret C, Samson J, Liyakat Ali TM, et al. A primate-specific retroviral enhancer wires the XACT lncRNA into the core pluripotency network in humans. Nat Commun. 2019;10:5652. 31827084 10.1038/s41467-019-13551-1 PMC6906429

[14] Laporte M, Le Luyer J, Rougeux C, Dion-Côté AM, Krick M, Bernatchez L. DNA methylation reprogramming, TE derepression, and postzygotic isolation of nascent animal species. Sci Adv. 2019;5(10):eaaw1644. 31663013 10.1126/sciadv.aaw1644 PMC6795504

[15] Zhao P, Peng C, Fang L, Wang Z, Liu GE. Taming transposable elements in livestock and poultry: A review of their roles and applications. Genet Sel Evol. 2023;55:50. 37479995 10.1186/s12711-023-00821-2 PMC10362595

[16] Kojima S, Koyama S, Ka M, Saito Y, Parrish EH, Endo M, et al. Mobile element variation contributes to population-specific genome diversification, gene regulation and disease risk. Nat Genet. 2023;55(6):939–51. 37169872 10.1038/s41588-023-01390-2

[17] Liang D, Zhao P, Si J, Fang L, Pairo-Castineira E, Hu X, et al. Genomic analysis revealed a convergent evolution of LINE-1 in coat color: A case study in water buffaloes (*Bubalus bubalis*). Mol Biol Evol. 2021;38(3):1122–36. 33212507 10.1093/molbev/msaa279 PMC7947781

[18] Yang J, Huang L, Yang M, Fan Y, Li L, Fang S, et al. Possible introgression of the *VRTN* mutation increasing vertebral number, carcass length and teat number from Chinese pigs into European pigs. Sci Rep. 2016;6:19240. 26781738 10.1038/srep19240 PMC4726066

[19] Guo X, Zhao C, Yang R, Wang Y, Hu X. Abcd4 is associated with mammary gland development in mammals. BMC Genomics. 2024;25:494. 38764031 10.1186/s12864-024-10398-9 PMC11103957

[20] Ren DR, Ren J, Ruan GF, Guo YM, Wu LH, Yang GC, et al. Mapping and fine mapping of quantitative trait loci for the number of vertebrae in a White Duroc × Chinese Erhualian intercross resource population. Anim Genet. 2012;43(5):545–51. 22497517 10.1111/j.1365-2052.2011.02313.x

[21] Mikawa S, Sato S, Nii M, Morozumi T, Yoshioka G, Imaeda N, et al. Identification of a second gene associated with variation in vertebral number in domestic pigs. BMC Genet. 2011;12:5. 21232157 10.1186/1471-2156-12-5 PMC3024977

[22] Chen C, Zheng Y, Wang M, Murani E, D’Alessandro E, Moawad AS, et al. Sine insertion in the intron of pig ghr may decrease its expression by acting as a repressor. Animals (Basel). 2021;11(7):1871. 34201672 10.3390/ani11071871 PMC8300111

[23] Teng J, Gao Y, Yin H, Bai Z, Liu S, Zeng H, et al. A compendium of genetic regulatory effects across pig tissues. Nat Genet. 2024;56(1):112–23. 38177344 10.1038/s41588-023-01585-7 PMC10786720

[24] Yang R, Guo X, Zhu D, Tan C, Bian C, Ren J, et al. Accelerated deciphering of the genetic architecture of agricultural economic traits in pigs using a low-coverage whole-genome sequencing strategy. Gigascience. 2021;10(7):giab048. 34282453 10.1093/gigascience/giab048 PMC8290195

[25] Tong X, Chen D, Hu J, Lin S, Ling Z, Ai H, et al. Accurate haplotype construction and detection of selection signatures enabled by high quality pig genome sequences. Nat Commun. 2023;14:5126. 37612277 10.1038/s41467-023-40434-3 PMC10447580

[26] Yang H, Wu J, Huang X, Zhou Y, Zhang Y, Liu M, et al. Abo genotype alters the gut microbiota by regulating galnac levels in pigs. Nature. 2022;606(7913):358–67. 35477154 10.1038/s41586-022-04769-z PMC9157047

[27] Ai H, Fang X, Yang B, Huang Z, Chen H, Mao L, et al. Adaptation and possible ancient interspecies introgression in pigs identified by whole-genome sequencing. Nat Genet. 2015;47(3):217–25. 25621459 10.1038/ng.3199

[28] Ai H, Zhang M, Yang B, Goldberg A, Li W, Ma J, et al. Human-mediated admixture and selection shape the diversity on the modern swine (*Sus scrofa*) Y chromosomes. Mol Biol Evol. 2021;38(11):5051–65. 34343337 10.1093/molbev/msab230 PMC8557463

[29] Chen H, Huang M, Yang B, Wu Z, Deng Z, Hou Y, et al. Introgression of Eastern Chinese and Southern Chinese haplotypes contributes to the improvement of fertility and immunity in European modern pigs. Gigascience. 2020;9(3):giaa014. 32141510 10.1093/gigascience/giaa014 PMC7059266

[30] Zhang M, Yang Q, Ai H, Huang L. Revisiting the evolutionary history of pigs via de novo mutation rate estimation in a three-generation pedigree. Genomics Proteomics Bioinformatics. 2022;20(6):1040–52. 35181533 10.1016/j.gpb.2022.02.001 PMC10225487

[31] Chen S, Zhou Y, Chen Y, Gu J. Fastp: An ultra-fast all-in-one fastq preprocessor. Bioinformatics. 2018;34(17):i884–90. 30423086 10.1093/bioinformatics/bty560 PMC6129281

[32] Warr A, Affara N, Aken B, Beiki H, Bickhart DM, Billis K, et al. An improved pig reference genome sequence to enable pig genetics and genomics research. Gigascience. 2020;9(6):giaa051. 32543654 10.1093/gigascience/giaa051 PMC7448572

[33] Li H, Durbin R. Fast and accurate short read alignment with burrows-wheeler transform. Bioinformatics. 2009;25(14):1754–60. 19451168 10.1093/bioinformatics/btp324 PMC2705234

[34] Pedersen BS, Quinlan AR. Mosdepth: Quick coverage calculation for genomes and exomes. Bioinformatics. 2018;34(5):867–8. 29096012 10.1093/bioinformatics/btx699 PMC6030888

[35] Li H, Handsaker B, Wysoker A, Fennell T, Ruan J, Homer N, et al. The sequence alignment/map format and samtools. Bioinformatics. 2009;25(16):2078–9. 19505943 10.1093/bioinformatics/btp352 PMC2723002

[36] Flynn JM, Hubley R, Goubert C, Rosen J, Clark AG, Feschotte C, et al. Repeatmodeler2 for automated genomic discovery of transposable element families. Proc Natl Acad Sci U S A. 2020;117(17):9451–7. 32300014 10.1073/pnas.1921046117 PMC7196820

[37] Rimmer A, Phan H, Mathieson I, Iqbal Z, Twigg SRF, Wilkie AOM, et al. Integrating mapping-, assembly- and haplotype-based approaches for calling variants in clinical sequencing applications. Nat Genet. 2014;46(8):912–8. 25017105 10.1038/ng.3036 PMC4753679

[38] Chang CC, Chow CC, Tellier LC, Vattikuti S, Purcell SM, Lee JJ. Second-generation plink: Rising to the challenge of larger and richer datasets. Gigascience. 2015;4:7. 25722852 10.1186/s13742-015-0047-8 PMC4342193

[39] Kern C, Wang Y, Xu X, Pan Z, Halstead M, Chanthavixay G, et al. Functional annotations of three domestic animal genomes provide vital resources for comparative and agricultural research. Nat Commun. 2021;12:1821. 33758196 10.1038/s41467-021-22100-8 PMC7988148

[40] Quinlan AR, Hall IM. Bedtools: A flexible suite of utilities for comparing genomic features. Bioinformatics. 2010;26(6):841–2. 20110278 10.1093/bioinformatics/btq033 PMC2832824

[41] Lefort V, Desper R, Gascuel O. Fastme 2.0: A comprehensive, accurate, and fast distance-based phylogeny inference program. Mol Biol Evol. 2015;32(10):2798–800. 26130081 10.1093/molbev/msv150 PMC4576710

[42] Letunic I, Bork P. Interactive tree of life (itol) v5: An online tool for phylogenetic tree display and annotation. Nucleic Acids Res. 2021;49(W1):W293-w296. 33885785 10.1093/nar/gkab301 PMC8265157

[43] Danecek P, Auton A, Abecasis G, Albers CA, Banks E, DePristo MA, et al. The variant call format and vcftools. Bioinformatics. 2011;27(15):2156–8. 21653522 10.1093/bioinformatics/btr330 PMC3137218

[44] Kim D, Paggi JM, Park C, Bennett C, Salzberg SL. Graph-based genome alignment and genotyping with HISAT2 and HISAT-genotype. Nat Biotechnol. 2019;37:907–15. 31375807 10.1038/s41587-019-0201-4 PMC7605509

[45] Liao Y, Smyth GK, Shi W. Featurecounts: An efficient general purpose program for assigning sequence reads to genomic features. Bioinformatics. 2014;30(7):923–30. 24227677 10.1093/bioinformatics/btt656

[46] Li YI, Knowles DA, Humphrey J, Barbeira AN, Dickinson SP, Im HK, et al. Annotation-free quantification of RNA splicing using leafcutter. Nat Genet. 2018;50(1):151–8. 29229983 10.1038/s41588-017-0004-9 PMC5742080

[47] Ongen H, Buil A, Brown AA, Dermitzakis ET, Delaneau O. Fast and efficient qtl mapper for thousands of molecular phenotypes. Bioinformatics. 2016;32(10):1479–85. 26708335 10.1093/bioinformatics/btv722 PMC4866519

[48] Benner C, Spencer CC, Havulinna AS, Salomaa V, Ripatti S, Pirinen M. Finemap: Efficient variable selection using summary data from genome-wide association studies. Bioinformatics. 2016;32(10):1493–501. 26773131 10.1093/bioinformatics/btw018 PMC4866522

[49] Danecek P, Bonfield JK, Liddle J, Marshall J, Ohan V, Pollard MO, et al. Twelve years of samtools and bcftools. Gigascience. 2021;10(2):giab008. 33590861 10.1093/gigascience/giab008 PMC7931819

[50] Hofmeister RJ, Ribeiro DM, Rubinacci S, Delaneau O. Accurate rare variant phasing of whole-genome and whole-exome sequencing data in the UK Biobank. Nat Genet. 2023;55:1243–9. 37386248 10.1038/s41588-023-01415-w PMC10335929

[51] Das S, Forer L, Schönherr S, Sidore C, Locke AE, Kwong A, et al. Next-generation genotype imputation service and methods. Nat Genet. 2016;48:1284–7. 27571263 10.1038/ng.3656 PMC5157836

[52] Zhou X, Stephens M. Genome-wide efficient mixed-model analysis for association studies. Nat Genet. 2012;44:821–4. 22706312 10.1038/ng.2310 PMC3386377

[53] Zong W, Zhao R, Wang X, Zhou C, Wang J, Chen C, et al. Population genetic analysis based on the polymorphisms mediated by transposons in the genomes of pig. DNA Res. 2024;31(2):dsae008. 38447059 10.1093/dnares/dsae008 PMC11090087

[54] Berthelier J, Furci L, Asai S, Sadykova M, Shimazaki T, Shirasu K, et al. Long-read direct RNA sequencing reveals epigenetic regulation of chimeric gene-transposon transcripts in arabidopsis thaliana. Nat Commun. 2023;14:3248. 37277361 10.1038/s41467-023-38954-z PMC10241880

[55] Payer LM, Burns KH. Transposable elements in human genetic disease. Nat Rev Genet. 2019;20(12):760–72. 31515540 10.1038/s41576-019-0165-8

[56] Huh MS, Price O’Dea T, Ouazia D, McKay BC, Parise G, Parks RJ, et al. Compromised genomic integrity impedes muscle growth after *Atrx* inactivation. J Clin Invest. 2012;122(12):4412–23. 23114596 10.1172/JCI63765 PMC3533543

[57] Huh MS, Young KG, Yan K, Price-O’Dea T, Picketts DJ. Recovery from impaired muscle growth arises from prolonged postnatal accretion of myonuclei in *Atrx* mutant mice. PLoS ONE. 2017;12(11):e0186989. 29095838 10.1371/journal.pone.0186989 PMC5667798

[58] Faggion S, Boschi E, Veroneze R, Carnier P, Bonfatti V. Genomic prediction and genome-wide association study for boar taint compounds. Animals (Basel). 2023;13(15):2450. 37570259 10.3390/ani13152450 PMC10417264

[59] Wang Y, Zhang C, Peng Y, Cai X, Hu X, Bosse M, et al. Whole-genome analysis reveals the hybrid formation of Chinese indigenous DHB pig following human migration. Evol Appl. 2022;15(3):501–14. 35386394 10.1111/eva.13366 PMC8965386

[60] Saini T, Chauhan A, Ahmad SF, Kumar A, Vaishnav S, Singh S, et al. Elucidation of population stratifying markers and selective sweeps in crossbred landlly pig population using genome-wide SNP data. Mamm Genome. 2024;35(2):170–85. 38485788 10.1007/s00335-024-10029-4

[61] Yan G, Liu X, Xiao S, Xin W, Xu W, Li Y, et al. An imputed whole-genome sequence-based gwas approach pinpoints causal mutations for complex traits in a specific swine population. Sci China Life Sci. 2022;65(4):781–94. 34387836 10.1007/s11427-020-1960-9

[62] Derks MFL, Gross C, Lopes MS, Reinders MJT, Bosse M, Gjuvsland AB, et al. Accelerated discovery of functional genomic variation in pigs. Genomics. 2021;113(4):2229–39. 34022350 10.1016/j.ygeno.2021.05.017

[63] Lee J, Mun S, Kim DH, Cho CS, Oh DY, Han K. Chicken (*Gallus gallus*) endogenous retrovirus generates genomic variations in the chicken genome. Mob DNA. 2017;8:2. 28138342 10.1186/s13100-016-0085-5 PMC5260121

[64] Wang X, D’Alessandro E, Chi C, Moawad AS, Zong W, Chen C, et al. Genetic evaluation and population structure of Jiangsu native pigs in China revealed by sine insertion polymorphisms. Animals (Basel). 2022;12(11):1345. 35681812 10.3390/ani12111345 PMC9179424

[65] Chessa B, Pereira F, Arnaud F, Amorim A, Goyache F, Mainland I, et al. Revealing the history of sheep domestication using retrovirus integrations. Science. 2009;324(5926):532–6. 19390051 10.1126/science.1170587 PMC3145132

[66] Yang L, Yin H, Bai L, Yao W, Tao T, Zhao Q, et al. Mapping and functional characterization of structural variation in 1060 pig genomes. Genome Biol. 2024;25(1):116. 38715020 10.1186/s13059-024-03253-3 PMC11075355

[67] Chen C, D’Alessandro E, Murani E, Zheng Y, Giosa D, Yang N, et al. Sine jumping contributes to large-scale polymorphisms in the pig genomes. Mob DNA. 2021;12(1):17. 34183049 10.1186/s13100-021-00246-y PMC8240389

[68] Zhao P, Gu L, Gao Y, Pan Z, Liu L, Li X, et al. Young sines in pig genomes impact gene regulation, genetic diversity, and complex traits. Commun Biol. 2023;6(1):894. 37652983 10.1038/s42003-023-05234-x PMC10471783

[69] Li Z, Liu X, Wang C, Li Z, Jiang B, Zhang R, et al. The pig pangenome provides insights into the roles of coding structural variations in genetic diversity and adaptation. Genome Res. 2023;33(10):1833–47. 37914227 10.1101/gr.277638.122 PMC10691484

[70] Zong W, Wang J, Zhao R, Niu N, Su Y, Hu Z, et al. Associations of genome-wide structural variations with phenotypic differences in cross-bred eurasian pigs. J Anim Sci Biotechnol. 2023;14:136. 37805653 10.1186/s40104-023-00929-x PMC10559557

[71] Frazer KA, Ballinger DG, Cox DR, Hinds DA, Stuve LL, Gibbs RA, et al. A second generation human haplotype map of over 3.1 million SNPs. Nature. 2007;449(7164):851–61. 17943122 10.1038/nature06258 PMC2689609

[72] Auton A, Brooks LD, Durbin RM, Garrison EP, Kang HM, Korbel JO, et al. A global reference for human genetic variation. Nature. 2015;526(7571):68–74. 26432245 10.1038/nature15393 PMC4750478

[73] Wu Z, Gong H, Zhang M, Tong X, Ai H, Xiao S, et al. A worldwide map of swine short tandem repeats and their associations with evolutionary and environmental adaptations. Genet Sel Evol. 2021;53:39. 33892623 10.1186/s12711-021-00631-4 PMC8063339

[74] Trizzino M, Park Y, Holsbach-Beltrame M, Aracena K, Mika K, Caliskan M, et al. Transposable elements are the primary source of novelty in primate gene regulation. Genome Res. 2017;27(10):1623–33. 28855262 10.1101/gr.218149.116 PMC5630026

[75] Jacques P, Jeyakani J, Bourque G. The majority of primate-specific regulatory sequences are derived from transposable elements. PLoS Genet. 2013;9(5):e1003504. 23675311 10.1371/journal.pgen.1003504 PMC3649963

[76] Zhao P, Du H, Jiang L, Zheng X, Feng W, Diao C, et al. Pre-1 revealed previous unknown introgression events in eurasian boars during the middle pleistocene. Genome Biol Evol. 2020;12(10):1751–64. 33151306 10.1093/gbe/evaa142 PMC7643367

[77] Chen C, Wang X, Zong W, D’Alessandro E, Giosa D, Guo Y, et al. Genetic diversity and population structures in Chinese miniature pigs revealed by sine retrotransposon insertion polymorphisms, a new type of genetic markers. Animals (Basel). 2021;11(4):1136. 33921134 10.3390/ani11041136 PMC8071531

[78] Cao X, Zhang Y, Payer LM, Lords H, Steranka JP, Burns KH, et al. Polymorphic mobile element insertions contribute to gene expression and alternative splicing in human tissues. Genome Biol. 2020;21(1):185. 32718348 10.1186/s13059-020-02101-4 PMC7385971

[79] Miao B, Fu S, Lyu C, Gontarz P, Wang T, Zhang B. Tissue-specific usage of transposable element-derived promoters in mouse development. Genome Biol. 2020;21(1):255. 32988383 10.1186/s13059-020-02164-3 PMC7520981

[80] Villanueva-Cañas JL, Horvath V, Aguilera L, González J. Diverse families of transposable elements affect the transcriptional regulation of stress-response genes in drosophila melanogaster. Nucleic Acids Res. 2019;47(13):6842–57. 31175824 10.1093/nar/gkz490 PMC6649756

[81] Cohen CJ, Lock WM, Mager DL. Endogenous retroviral ltrs as promoters for human genes: A critical assessment. Gene. 2009;448(2):105–14. 19577618 10.1016/j.gene.2009.06.020

[82] Lanciano S, Cristofari G. Measuring and interpreting transposable element expression. Nat Rev Genet. 2020;21(12):721–36. 32576954 10.1038/s41576-020-0251-y

[83] Macfarlan TS, Gifford WD, Driscoll S, Lettieri K, Rowe HM, Bonanomi D, et al. Embryonic stem cell potency fluctuates with endogenous retrovirus activity. Nature. 2012;487(7405):57–63. 22722858 10.1038/nature11244 PMC3395470

[84] Yang F, Huang X, Zang R, Chen J, Fidalgo M, Sanchez-Priego C, et al. DUX-miR-344-ZMYM2-mediated activation of MERVL LTRs induces a totipotent 2C-like state. Cell Stem Cell. 2020;26(2):234–50. 32032525 10.1016/j.stem.2020.01.004 PMC8074926

[85] Fueyo R, Judd J, Feschotte C, Wysocka J. Roles of transposable elements in the regulation of mammalian transcription. Nat Rev Mol Cell Biol. 2022;23(7):481–97. 35228718 10.1038/s41580-022-00457-y PMC10470143

[86] Jiang T, Zhou ZM, Ling ZQ, Zhang Q, Wu ZZ, Yang JW, et al. Pig H3K4me3, H3K27ac, and gene expression profiles reveal reproductive tissue-specific activity of transposable elements. Zool Res. 2024;45(1):138–51. 38155423 10.24272/j.issn.2095-8137.2023.060 PMC10839656

[87] Friedlander E, Steinrücken M. A numerical framework for genetic hitchhiking in populations of variable size. Genetics. 2022;220(3):iyac012. 35143667 10.1093/genetics/iyac012 PMC8893261

[88] Denner J. How active are porcine endogenous retroviruses (PERVs)? Viruses. 2016;8(8):215. 27527207 10.3390/v8080215 PMC4997577

[89] Chen JQ, Zhang MP, Tong XK, Li JQ, Zhang Z, Huang F, et al. Scan of the endogenous retrovirus sequences across the swine genome and survey of their copy number variation and sequence diversity among various Chinese and Western pig breeds. Zool Res. 2022;43(3):423–41. 35437972 10.24272/j.issn.2095-8137.2021.379 PMC9113972

[90] Denner J. The origin of porcine endogenous retroviruses (PERVs). Arch Virol. 2021;166(4):1007–13. 33547957 10.1007/s00705-020-04925-8

[91] Griffith B. Pig-to-human transplants take a leap toward reality. Nat Med. 2022;28(3):423. 10.1038/s41591-022-01770-x 35314821

[92] Denner J, Schuurman HJ. High prevalence of recombinant porcine endogenous retroviruses (PERV-A/Cs) in minipigs: A review on origin and presence. Viruses. 2021;13(9):1869. 34578447 10.3390/v13091869 PMC8473008

[93] Sexton CE, Han MV. Paired-end mappability of transposable elements in the human genome. Mob DNA. 2019;10:29. 31320939 10.1186/s13100-019-0172-5 PMC6617613

[94] Gardner EJ, Lam VK, Harris DN, Chuang NT, Scott EC, Pittard WS, et al. The mobile element locator tool (MELT): Population-scale mobile element discovery and biology. Genome Res. 2017;27(11):1916–29. 28855259 10.1101/gr.218032.116 PMC5668948

