# Supplementary material for: Construction of the porcine genome mobile element variations and investigation of its role in population diversity and gene expression
Source: J Anim Sci Biotechnol. 2024 Dec 4;15:162. doi: 10.1186/s40104-024-01121-5 (PMC11616153; doi:10.1186/s40104-024-01121-5)
Supplement: Supplementary file 1 — Additional file 1: Fig. S1. Assessment of MEVs genotyping accuracy. A) Concordance ratio of MEVs detected by Next-generation sequencing (NGS) and Third-generation sequencing (TGS) data for the three individuals. B) Mendelian genetic ratio of 12 trio families. Fig. S2. Characterization of MEVs in 747 pigs. A) The length distribution of four different types of MEVs. B) Cumulative distribution of MEVs, as the sample size increases in each group, the total number of MEV counts increases accordingly. And the MEV counts of each individual in each group, sorted in ascending order within the group. Fig. S3. Population genetic analysis using MEVs. A) Principal component analysis of all individuals. Different colors represent different populations. B) Neighbor-joining phylogenetic tree of pig breeds based on MEVs, with varieties represented by different colors consistent with the PCA diagram. The scale bar represents proportional to similarity. Fig. S4. Gene function enrichment analysis. A) Functional enrichment of 294 differential genes between Asian and European pigs. B) Functional enrichment of 291 differential genes between Asian domestic pigs and Asian wild pigs. Fig. S5. Breed-specific MEVs analysis. Heatmap showing breed specific MEVs that overlap with genes in all 11 breeds, and bar plot above heatmap represents number of breed-specific MEVs. Fig. S6. Distribution characteristics of eMEVs and sMEVs. A) The fold change of eMEVs at different genomic locations. B) The fold change of sMEVs at different genomic locations. C) The fold change of eMEVs at the four active epigenetic markers in liver tissue of pigs. D) The fold change of sMEVs at the four active epigenetic markers in liver tissue of pigs. Fig. S7. Gene enrichment with a posterior probability greater than 0.5. Functional enrichment of 531 genes with a posterior probability greater than 0.5. Fig. S8. eQTL and sQTL analysis of 34 tissues from pigGTEx data based on imputed MEVs. A) The number of MEV-eQTLs that are [file 40104_2024_1121_MOESM1_ESM.docx]

**Supplemental figures**


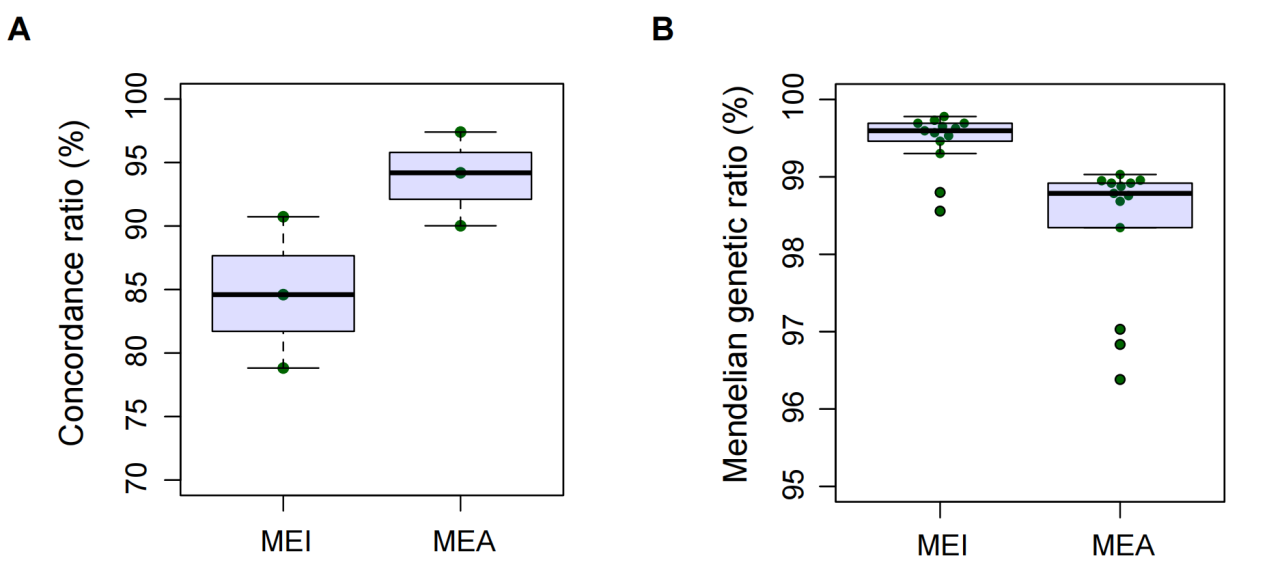


**Fig. S1 Assessment of MEVs genotyping accuracy
A)** Concordance ratio of MEVs detected by Next-generation sequencing (NGS) and Third-generation sequencing (TGS) data for the three individuals. (MEI: Mobile element insert; MEA: Mobile element absent).

**B)** Mendelian genetic ratio of 12 trio families.


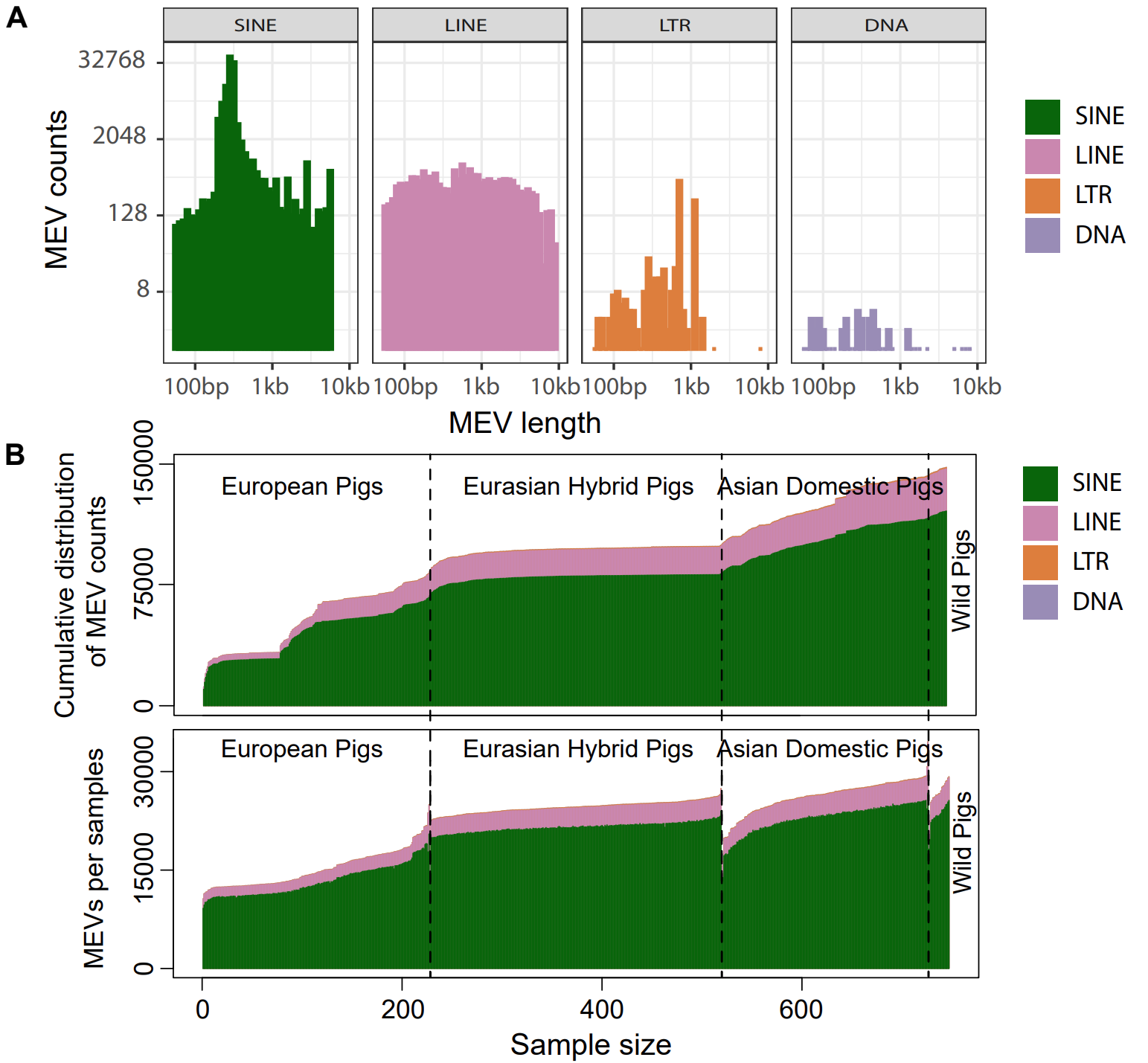


**Fig. S2 Characterization of MEVs in 747 pigs**

**A)** The length distribution of four different types of MEVs.
**B)** Cumulative distribution of MEVs, as the sample size increases in each group, the total number of MEV counts increases accordingly. And the MEV counts of each individual in each group, sorted in ascending order within the group.

**
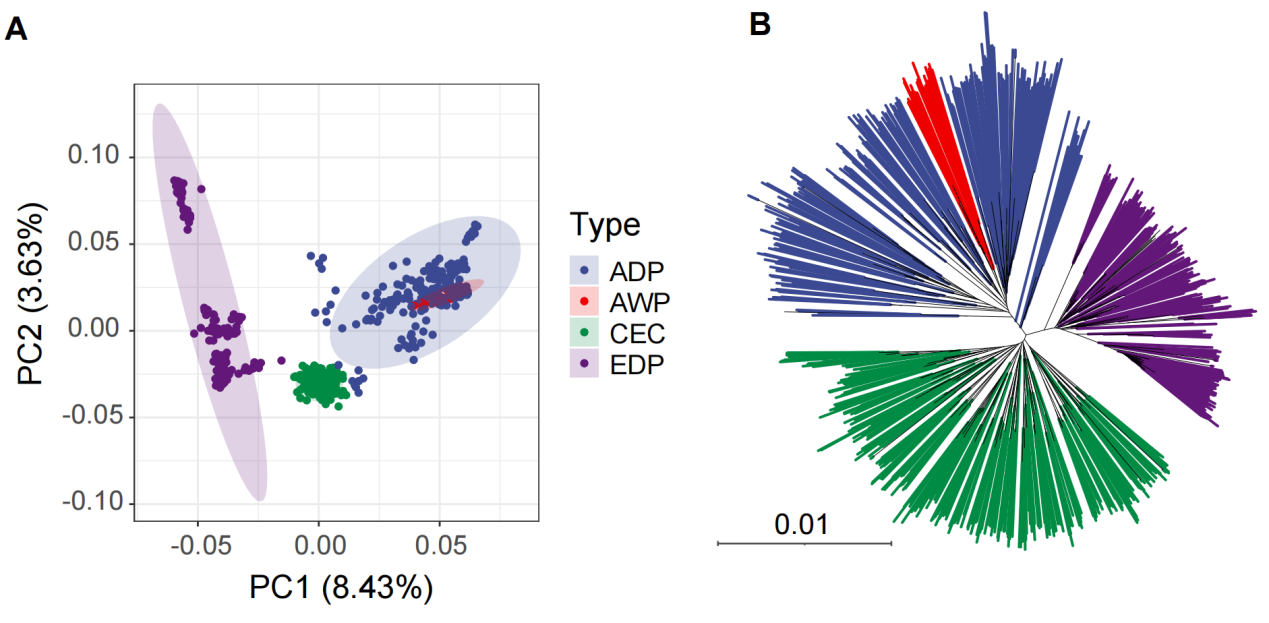
**

**Fig. S3 Population genetic analysis using MEVs**

**A)** Principal component analysis of all individuals. Different colors represent different populations.
**B)** Neighbor-joining phylogenetic tree of pig breeds based on MEVs, with varieties represented by different colors consistent with the PCA diagram. The scale bar represents proportional to similarity.

**Fig. S4 Gene function enrichment analysis**

**A)** Functional enrichment of 294 differential genes between Asian and European pigs.
**B)** Functional enrichment of 291 differential genes between Asian domestic pigs and Asian wild pigs.


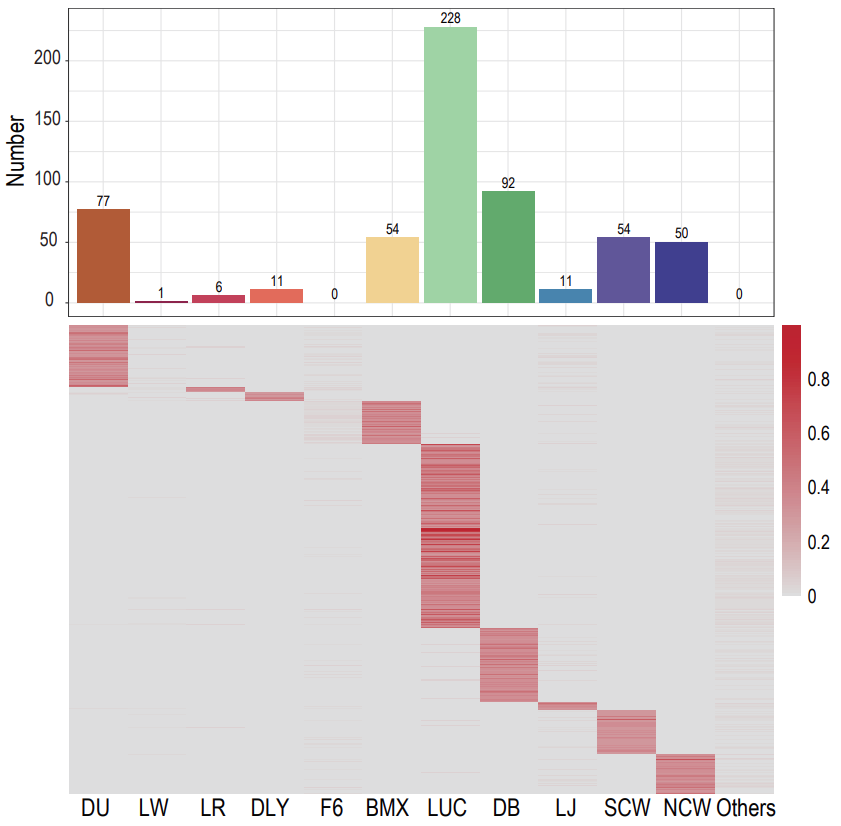


**Fig. S5 Breed-specific MEVs analysis**

Heatmap showing breed specific MEVs that overlap with genes in all 11 breeds, and bar plot above heatmap represents number of breed-specific MEVs. Duroc, DU; Large White pig, LW; Long White pig, LR; Du Chang Da, DLY; Hybrid sixth generation, F6; Bama Xiang pig, BMX; Luchuan pig, LUC; Debao pig, DB; Lijiang pig, LJ; Southern Chinese wild boar, SCW; Northern Chinese wild boar, NCW.


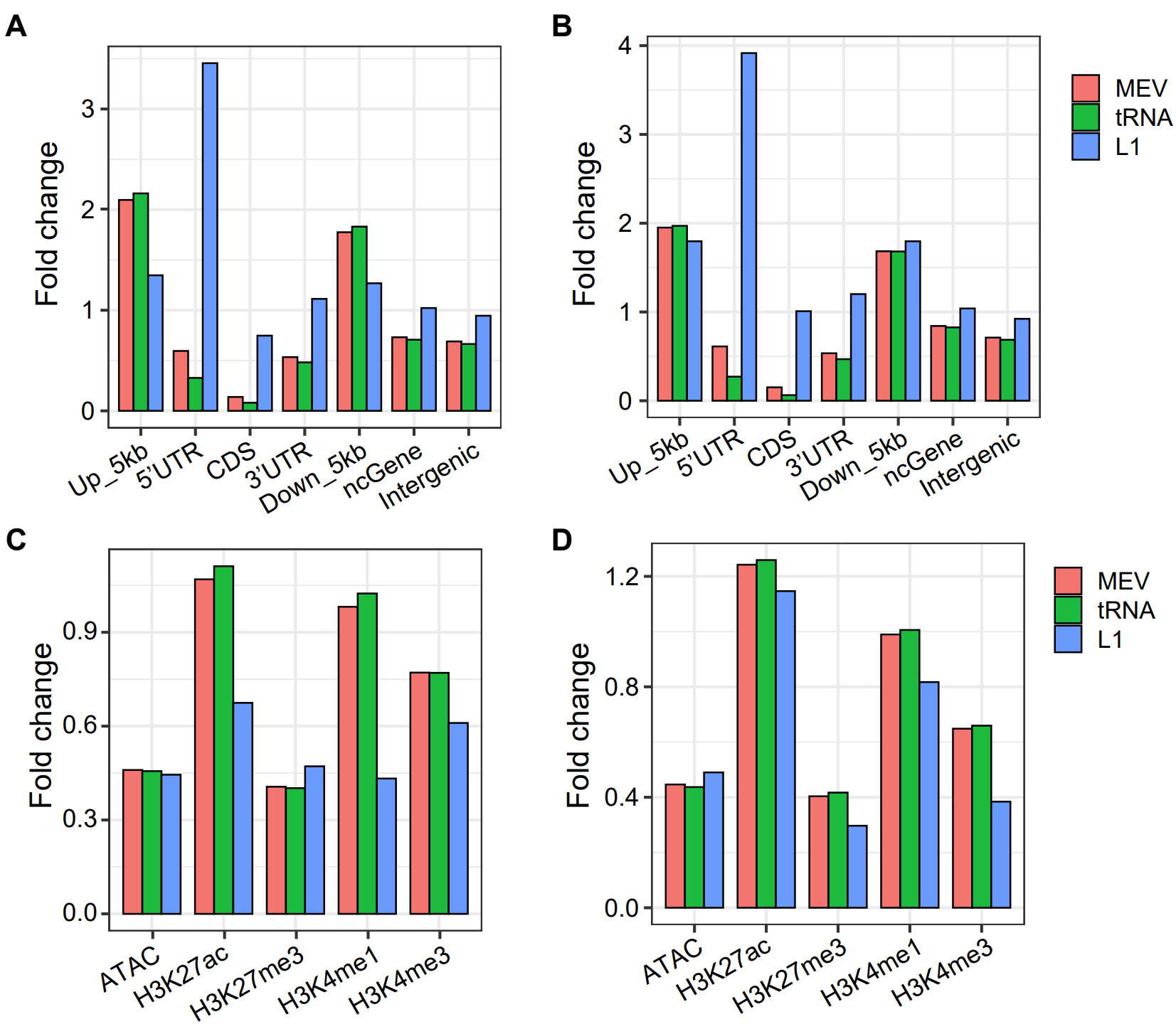


**Fig. S6 Distribution characteristics of eMEVs and sMEVs**

**A)** The fold change of eMEVs at different genomic locations.
**B)** The fold change of sMEVs at different genomic locations.

**C)** The fold change of eMEVs at the four active epigenetic markers in liver tissue of pigs.
**D)** The fold change of sMEVs at the four active epigenetic markers in liver tissue of pigs


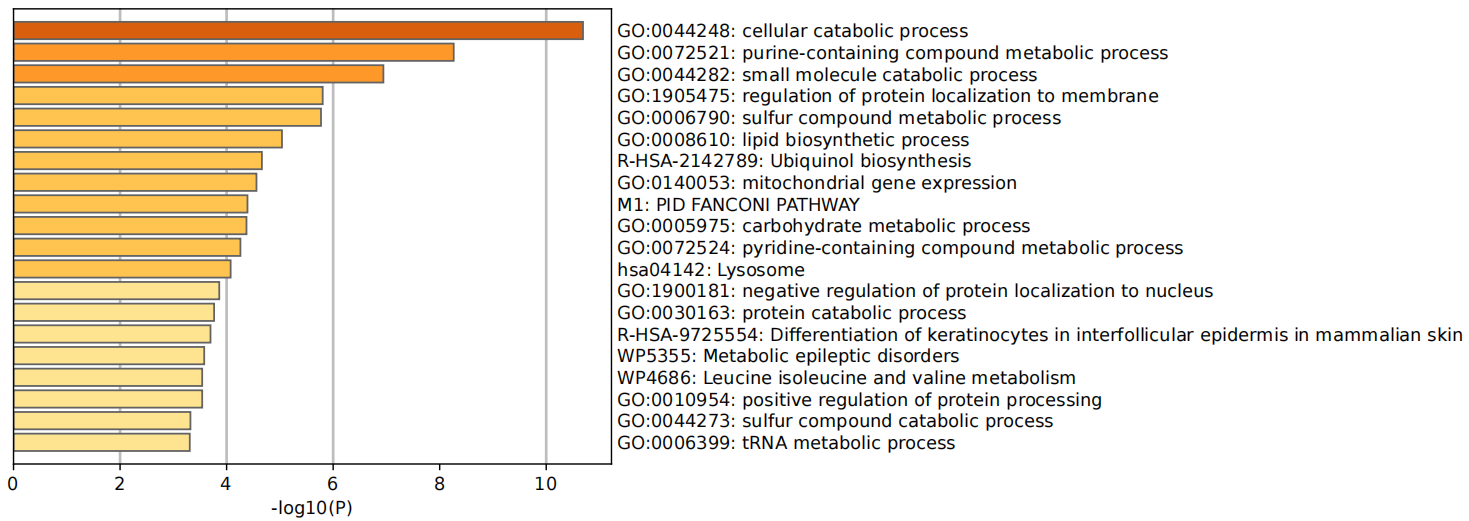


**Fig. S7 Gene enrichment with a posterior probability greater than 0.5**

Functional enrichment of 531 genes with a posterior probability greater than 0.5.


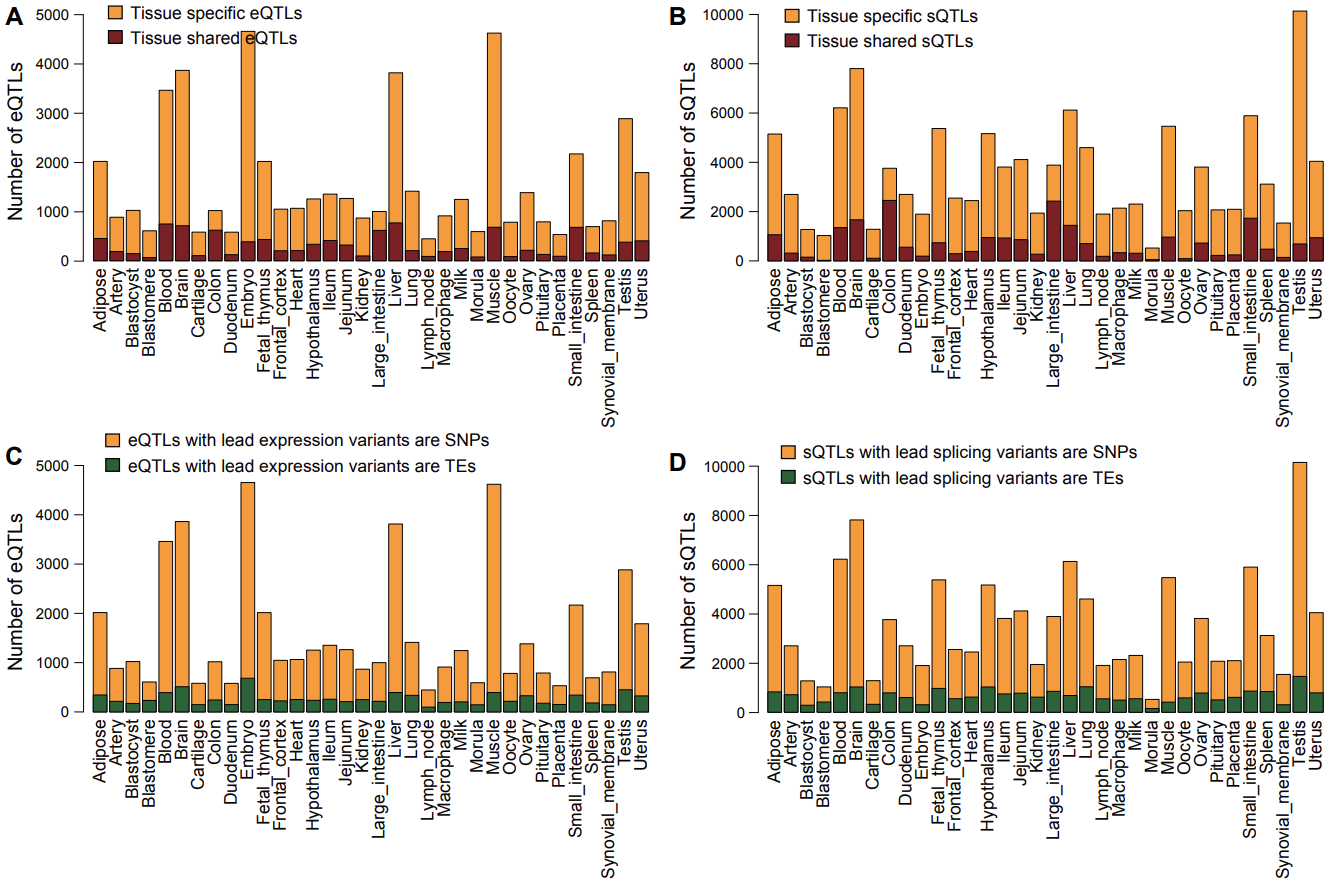


**Fig. S8** **eQTL and sQTL analysis of 34 tissues from pigGTEx data based on imputed MEVs.**
A) The number of MEV-eQTLs that are shared or specific between tissues.

B) The number of MEV-sQTLs that are shared or specific between tissues.

C) The MEV-eQTLs which lead expression variants are SNPs or MEVs.

D) The MEV-sQTLs which lead splicing variants are SNPs or MEVs.
